# Supplementary material for: Transposable element expression is associated with sex chromosome number in humans
Source: PLoS Genet. 2025 Jun 26;21(6):e1011668. doi: 10.1371/journal.pgen.1011668 (PMC12225981; doi:10.1371/journal.pgen.1011668)
Supplement: S1 File — (PDF) [file pgen.1011668.s001.pdf]

Transposable element expression is associated with sex chromosome number in humans

Jordan Teoli[1,2,3,4]*, Miriam Merenciano[2], Marie Fablet[2,5], Anamaria Necsulea[2], Daniel Siqueira-de-Oliveira[2], Alessandro Brandulas-Cammarata[6,7], Audrey Labalme[8], Hervé Lejeune[9], Jean-François Lemaitre[2], François Gueyffier[2], Damien Sanlaville[8], Claire Bardel[1,2], Cristina Vieira[2]¶, Gabriel AB Marais[2,4,10,11]¶, Ingrid Plotton[1,3,9]¶

[1] Laboratoire de Biochimie et Biologie Moléculaire, Centre de Biologie et Pathologie Est, Hospices Civils de Lyon; Bron, France.
[2] Université Claude Bernard Lyon 1, UMR CNRS 5558, Laboratoire de Biométrie et Biologie Evolutive ; Villeurbanne, France.
[3] Institut Cellule Souche et Cerveau (SBRI), Unité INSERM 1208, Centre de Recherche INSERM; Bron, France.
[4] CIBIO, Centro de Investigação em Biodiversidade e Recursos Genéticos, InBIO Laboratório Associado, Campus de Vairão, Universidade do Porto; Vairão, Portugal.
[5] Institut universitaire de France; Paris, France.
[6] Department of Ecology and Evolution, University of Lausanne; Lausanne, Switzerland.
[7] SIB Swiss Institute of Bioinformatics, Lausanne, Switzerland
[8] Service de Génétique, Hospices Civils de Lyon; Bron, France.
[9] Service de médecine de la reproduction, Hôpital Femme-Mère-Enfant, Hospices Civils de Lyon; Bron, France.
[10] Departamento de Biologia, Faculdade de Ciências, Universidade do Porto; Porto, Portugal.
[11] BIOPOLIS Program in Genomics, Biodiversity and Land Planning, CIBIO, Campus de Vairão; Vairão, Portugal.

¶Equal contributions as senior authors

* jordan.teoli@chu-lyon.fr

This S1 File includes:

Supplementary text (**Text A**)

Supplementary figures (**Fig A-V**)

Supplementary tables (**Table A-M**)

Other Supplementary Materials for this manuscript include the following:

Supplementary data (**S1-S7 Data**)

Supplementary text

Text A. Comparison of DEGs found in our study with those found in other studies

The differential expression study on genes found expected differentially expressed genes like those located on the Y chromosome to validate our protocol. Furthermore, we found *XIST* and *TSIX* genes to be expressed only in karyotypes containing two X chromosomes (47,XXY and 46,XX), which was also expected as these genes are implicated in the X inactivation occurring only in the presence of two or more X chromosomes [46–48]. In the karyotype with two or more X chromosomes (normal 46,XX karyotype or abnormal karyotype such as 47,XXY, 47,XXX…), the extra-X chromosomes are inactivated and that; only the X-linked genes escaping inactivation exhibit expression changes according to X chromosome dosage [73,77]. We found 6 of the 31 (19.4%) confirmed XCI escapees in the study of Wainer-Katsir et al. [74] among the upregulated genes retrieved when 47,XXY karyotype was compared to 46,XY karyotype. We also found differentially expressed genes when we compared 47,XXY to 46,XY or 46,XX karyotypes that were described elsewhere. Indeed, we shared 22 of the 130 (16.9%) upregulated genes (regardless if they are protein-coding or not) in 47,XXY compared to 46,XY reported in the study of Zhang et al. and 5 of the 111 (4.5%) downregulated genes in 47,XXY compared to 46,XY [73].

Supplementary figures

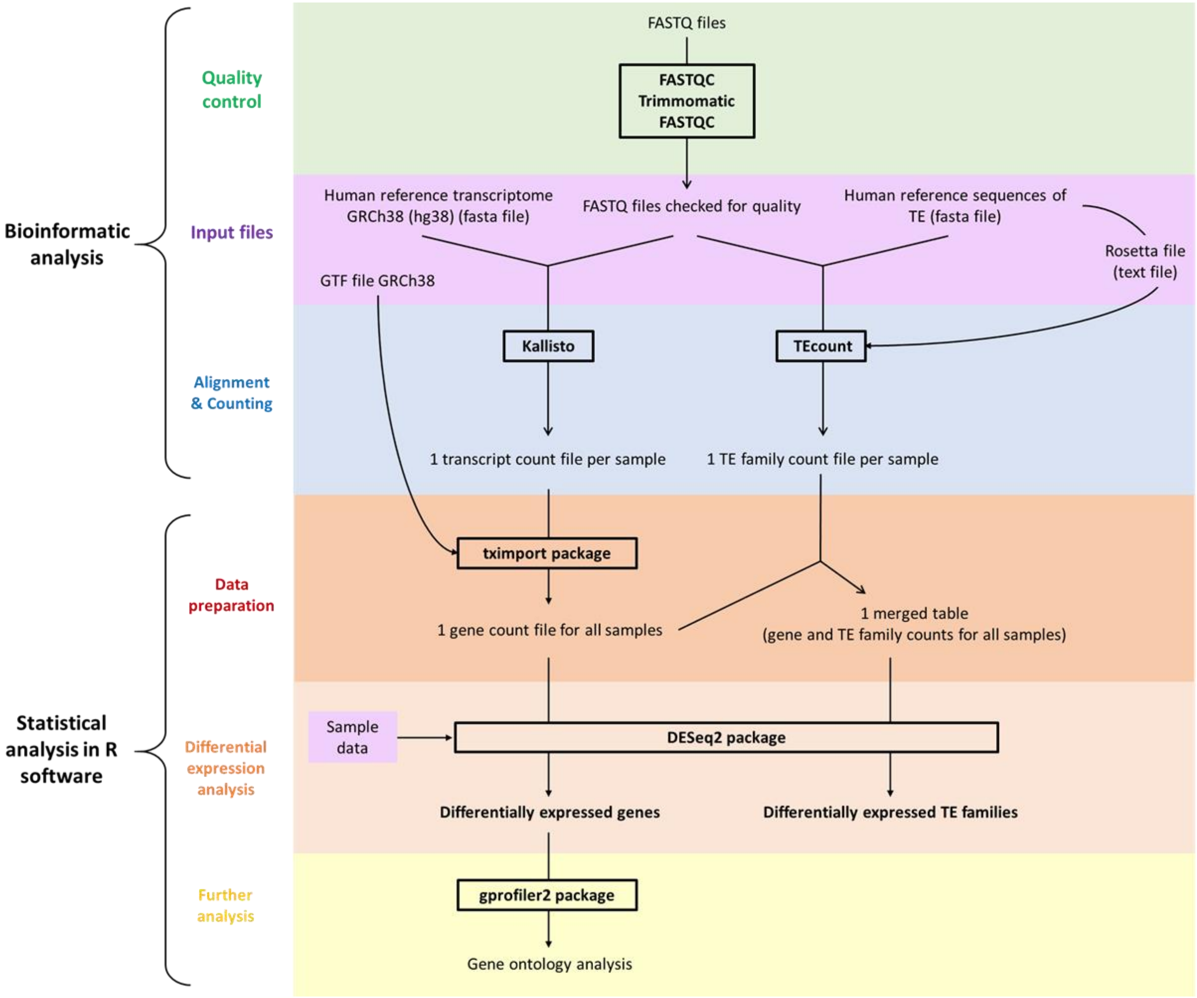

Fig A. Pipeline for gene and TE differential expression analysis.

GTF file: Gene transfer format file, TE: Transposable element.

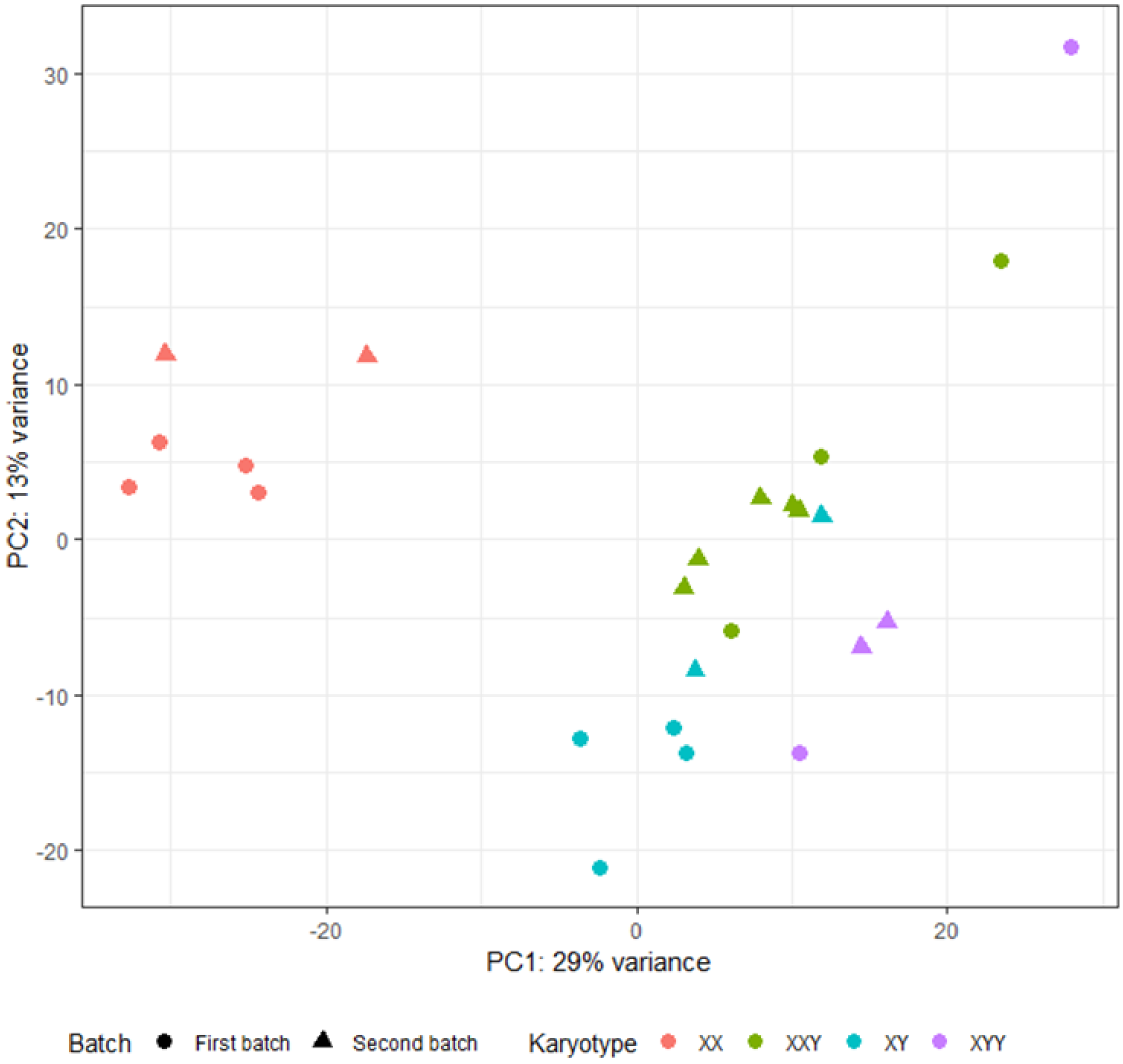

Fig B. Principal component analysis on the 500 genes explaining the most variance in the gonosome aneuploidy dataset after batch effect was removed.

Each dot represents one individual. Dots are colored according to karyotype (XX, XY, XXY, XYY) and shaped according to batch (first batch, second batch). Batch effect was removed before graphical display. PC: Principal Component.

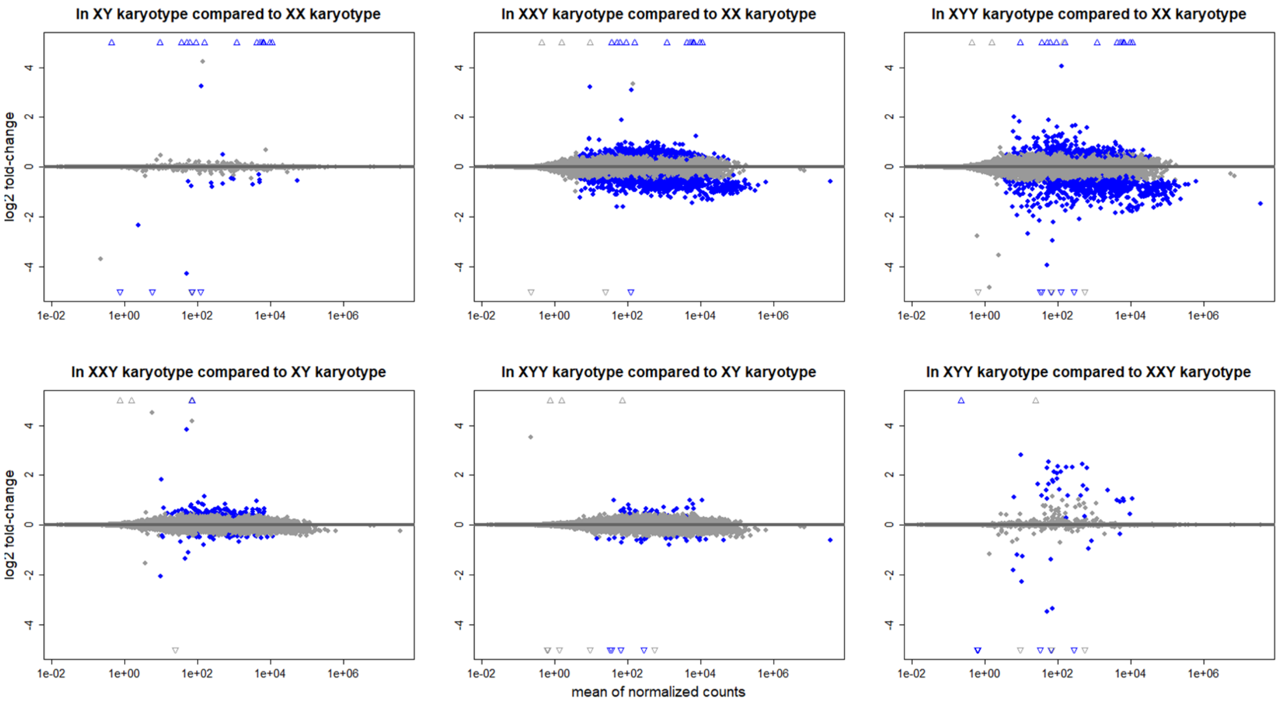

Fig C. MA plots on protein-coding genes in each pairwise karyotype comparison after batch adjustment in the gonosome aneuploidy dataset.

Minus-Average plots (MA plots) represent, for each protein-coding gene, the $\log_2$(normalized counts in group 1) – $\log_2$(normalized counts in group 2) on the y-axis and the mean expression (i.e. mean of normalized counts) across all the samples on the x-axis. Each dot or triangle represents one protein-coding gene. Blue dots show genes with an adjusted p-value < 0.05. Genes for which log2 fold-change is greater than 4 or less than -4 are represented by an empty triangle. Positive log2 fold-change means that the gene is upregulated in the first karyotype (e.g. XYY in the “XYY karyotype vs XX karyotype” comparison) compared to the second (e.g. XX in the “XYY karyotype vs XX karyotype” comparison). Negative log2 fold-change means that the gene is downregulated in the first karyotype (e.g. XYY in the “XYY karyotype vs XX karyotype” comparison) compared to the second (e.g. XX in the “XYY karyotype vs XX karyotype” comparison).

A

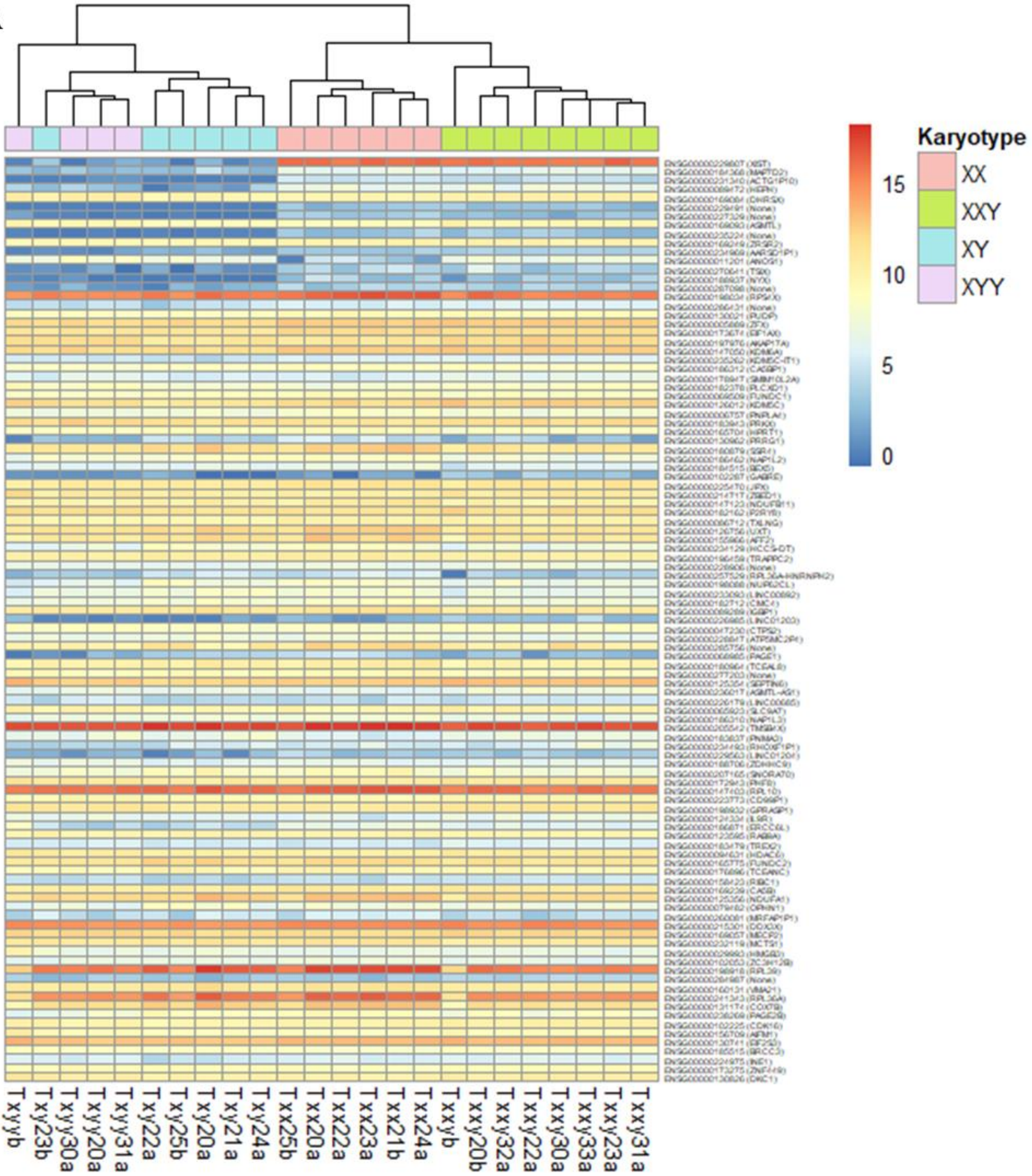
Karyotype
XX
XXY
XY
XYY
15
10
5
0
Txyyb
Txy23b
Txyy30a
Txyy20a
Txyy31a
Txy22a
Txy25b
Txy20a
Txy21a
Txy24a
Txx25b
Txx20a
Txx22a
Txx23a
Txx21b
Txx24a
Txxyb
Txxy20b
Txxy32a
Txxy22a
Txxy30a
Txxy33a
Txxy23a
Txxy31a

B

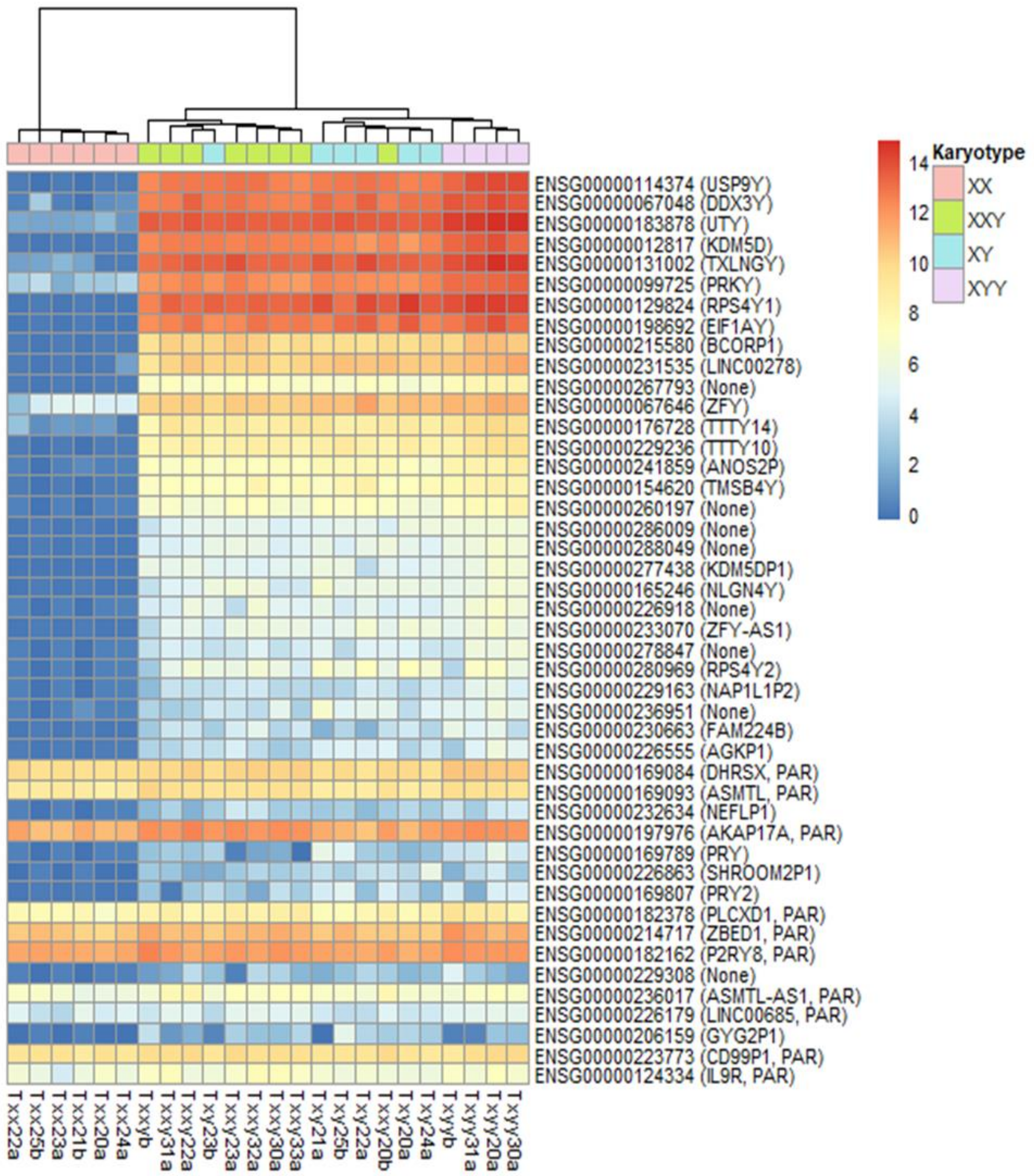
Karyotype
XX
XXY
XY
XYY
14
12
10
8
6
4
2
0
ENSG00000114374 (USP9Y)
ENSG00000067048 (DDX3Y)
ENSG00000183878 (UTY)
ENSG00000012817 (KDM5D)
ENSG00000131002 (TXLNGY)
ENSG00000099725 (PRKY)
ENSG00000129824 (RPS4Y1)
ENSG00000198692 (EIF1AY)
ENSG00000215580 (BCORP1)
ENSG00000231535 (LINC00278)
ENSG00000267793 (None)
ENSG00000067646 (ZFY)
ENSG00000176728 (TTTY14)
ENSG00000229236 (TTTY10)
ENSG00000241859 (ANOS2P)
ENSG00000154620 (TMSB4Y)
ENSG00000260197 (None)
ENSG00000286009 (None)
ENSG00000288049 (None)
ENSG00000277438 (KDM5DP1)
ENSG00000165246 (NLGN4Y)
ENSG00000226918 (None)
ENSG00000233070 (ZFY-AS1)
ENSG00000278847 (None)
ENSG00000280969 (RPS4Y2)
ENSG00000229163 (NAP1L1P2)
ENSG00000236951 (None)
ENSG00000230663 (FAM224B)
ENSG00000226555 (AGKP1)
ENSG00000169084 (DHRSX, PAR)
ENSG00000169093 (ASMTL, PAR)
ENSG00000232634 (NEFLP1)
ENSG00000197976 (AKAP17A, PAR)
ENSG00000169789 (PRY)
ENSG00000226863 (SHROOM2P1)
ENSG00000169807 (PRY2)
ENSG00000182378 (PLCXD1, PAR)
ENSG00000214717 (ZBED1, PAR)
ENSG00000182162 (P2RY8, PAR)
ENSG00000229308 (None)
ENSG00000236017 (ASMTL-AS1, PAR)
ENSG00000226179 (LINC00685, PAR)
ENSG00000206159 (GYG2P1)
ENSG00000223773 (CD99P1, PAR)
ENSG00000124334 (IL9R, PAR)
Txx22a
Txx25b
Txx23a
Txx21b
Txx20a
Txx24a
Txxyb
Txxy31a
Txxy22a
Txy23b
Txxy23a
Txxy32a
Txxy30a
Txxy33a
Txy21a
Txy25b
Txy22a
Txxy20b
Txy20a
Txy24a
Txyyb
Txyy31a
Txyy20a
Txyy30a

C

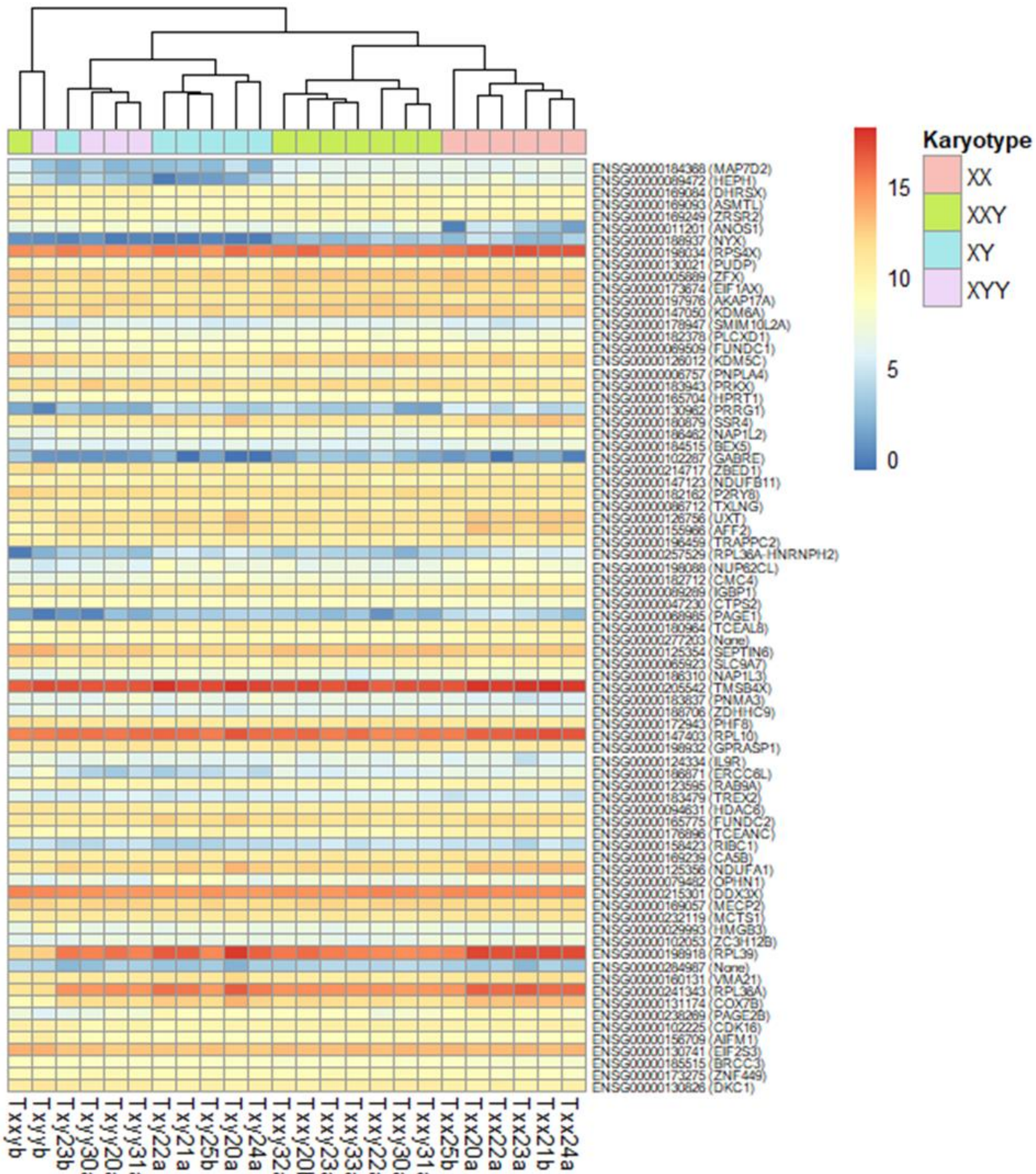
Karyotype
XX
XXY
XY
XYY
15
10
5
0
ENSG00000184368 (MAP7D2)
ENSG00000089472 (HEPH)
ENSG00000169084 (DHRSX)
ENSG00000169093 (ASMTL)
ENSG00000169249 (ZRSR2)
ENSG00000011201 (ANOS1)
ENSG00000188937 (NYX)
ENSG00000198034 (RPS4X)
ENSG00000130021 (PUDP)
ENSG00000005889 (ZFX)
ENSG00000173674 (EIF1AX)
ENSG00000197976 (AKAP17A)
ENSG00000147050 (KDM6A)
ENSG00000178947 (SMIM10L2A)
ENSG00000182378 (PLCXD1)
ENSG00000069509 (FUNDC1)
ENSG00000126012 (KDM5C)
ENSG00000006757 (PNPLA4)
ENSG00000183943 (PRKX)
ENSG00000165704 (HPRT1)
ENSG00000130962 (PRRG1)
ENSG00000180879 (SSR4)
ENSG00000186462 (NAP1L2)
ENSG00000184515 (BEX5)
ENSG00000102287 (GABRE)
ENSG00000214717 (ZBED1)
ENSG00000147123 (NDUFB11)
ENSG00000182162 (P2RY8)
ENSG00000086712 (TXLNG)
ENSG00000126756 (UXT)
ENSG00000155966 (AFF2)
ENSG00000196459 (TRAPPC2)
ENSG00000257529 (RPL36A-HNRNPH2)
ENSG00000198088 (NUP62CL)
ENSG00000182712 (CMC4)
ENSG00000089289 (IGBP1)
ENSG00000047230 (CTPS2)
ENSG00000068985 (PAGE1)
ENSG00000180964 (TCEAL8)
ENSG00000277203 (None)
ENSG00000125354 (SEPTIN6)
ENSG00000065923 (SLC9A7)
ENSG00000186310 (NAP1L3)
ENSG00000205542 (TMSB4X)
ENSG00000183837 (PNMA3)
ENSG00000188706 (ZDHHC9)
ENSG00000172943 (PHF8)
ENSG00000147403 (RPL10)
ENSG00000198932 (GPRASP1)
ENSG00000124334 (IL9R)
ENSG00000186871 (ERCC6L)
ENSG00000123595 (RAB9A)
ENSG00000183479 (TREX2)
ENSG00000094631 (HDAC6)
ENSG00000165775 (FUNDC2)
ENSG00000176896 (TCEANC)
ENSG00000158423 (RIBC1)
ENSG00000169239 (CA5B)
ENSG00000125356 (NDUFA1)
ENSG00000079482 (OPHN1)
ENSG00000215301 (DDX3X)
ENSG00000169057 (MECP2)
ENSG00000232119 (MCTS1)
ENSG00000029993 (HMGB3)
ENSG00000102053 (ZC3H12B)
ENSG00000198918 (RPL39)
ENSG00000284987 (None)
ENSG00000160131 (VMA21)
ENSG00000241343 (RPL36A)
ENSG00000131174 (COX7B)
ENSG00000238269 (PAGE2B)
ENSG00000102225 (CDK16)
ENSG00000156709 (AIFM1)
ENSG00000130741 (EIF2S3)
ENSG00000185515 (BRCC3)
ENSG00000173275 (ZNF449)
ENSG00000130826 (DKC1)
Txxyb
Txyyb
Txy23b
Txyy30a
Txyy20a
Txyy31a
Txy22a
Txy21a
Txy25b
Txy20a
Txy24a
Txxy32a
Txxy20b
Txxy23a
Txxy33a
Txxy22a
Txxy30a
Txxy31a
Txx25b
Txx20a
Txx22a
Txx23a
Txx21b
Txx24a

D

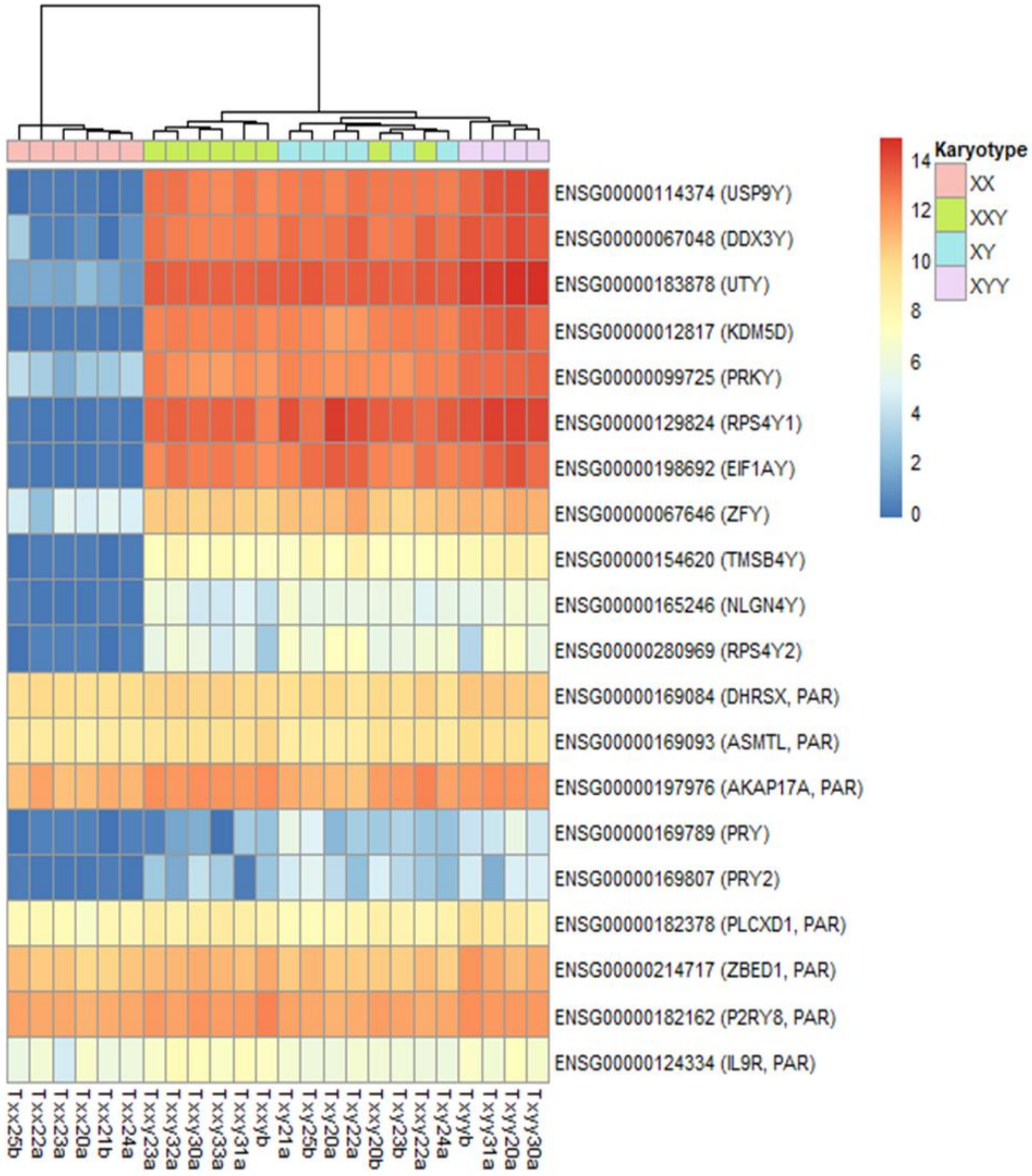

Fig D. Heatmaps of the differentially expressed genes after batch effect was removed in the gonosome aneuploidy dataset, first considering all genes (A) on the X chromosome (number of genes = 103) and (B) on the Y chromosome (number of genes = 45), and then considering only protein-coding genes (C) on the X chromosome (number of genes = 77) and (D) on the Y chromosome (number of genes = 20).

PAR indicates the genes located on the pseudo-autosomal region of the Y chromosome. Differentially expressed genes were obtained using the Likelihood-ratio test (see **Materials and Methods**) and the list of differentially expressed genes used in each heatmap is available in **S1 Data**. Batch effect was removed before graphical display.

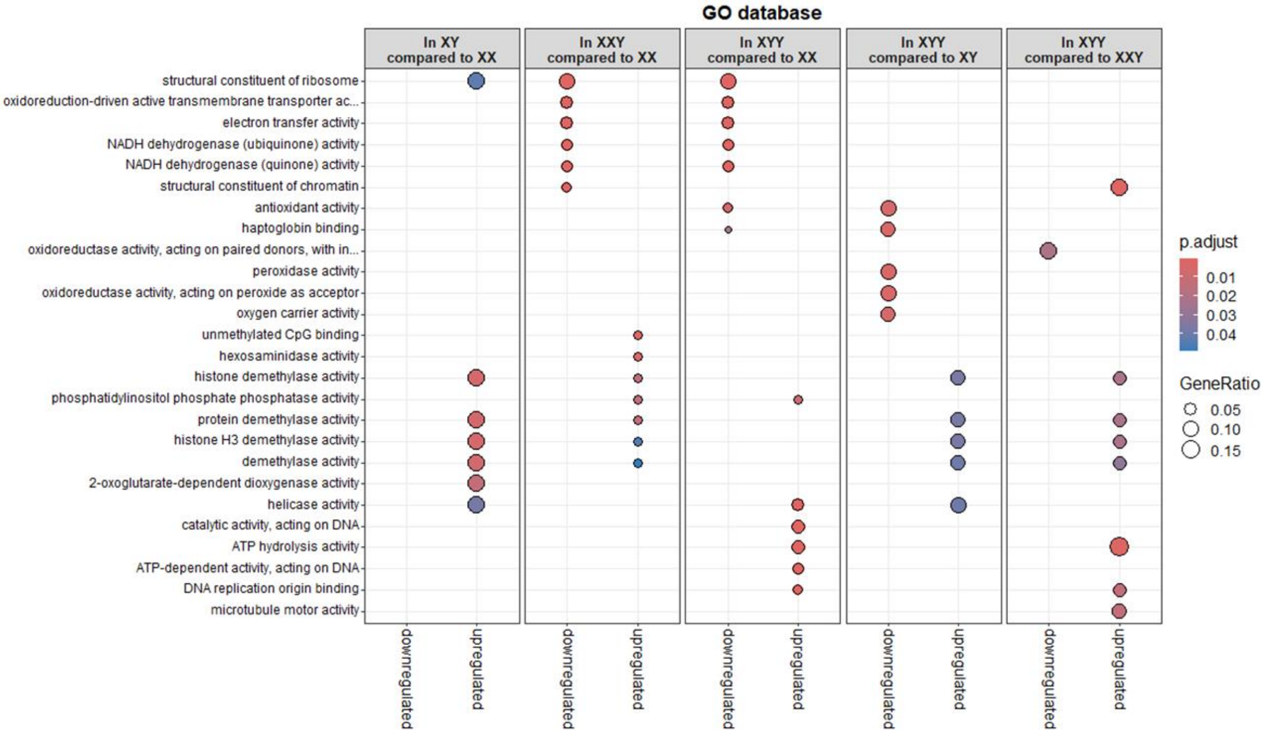

Fig E. Gene ontology analysis performed using the differentially expressed genes (regardless if they are protein-coding or not) and the GO database.

The plot shows the 5 most significant GO terms and shared GO terms considering downregulated genes and upregulated genes separately obtained from each pairwise karyotype comparison (when applicable).

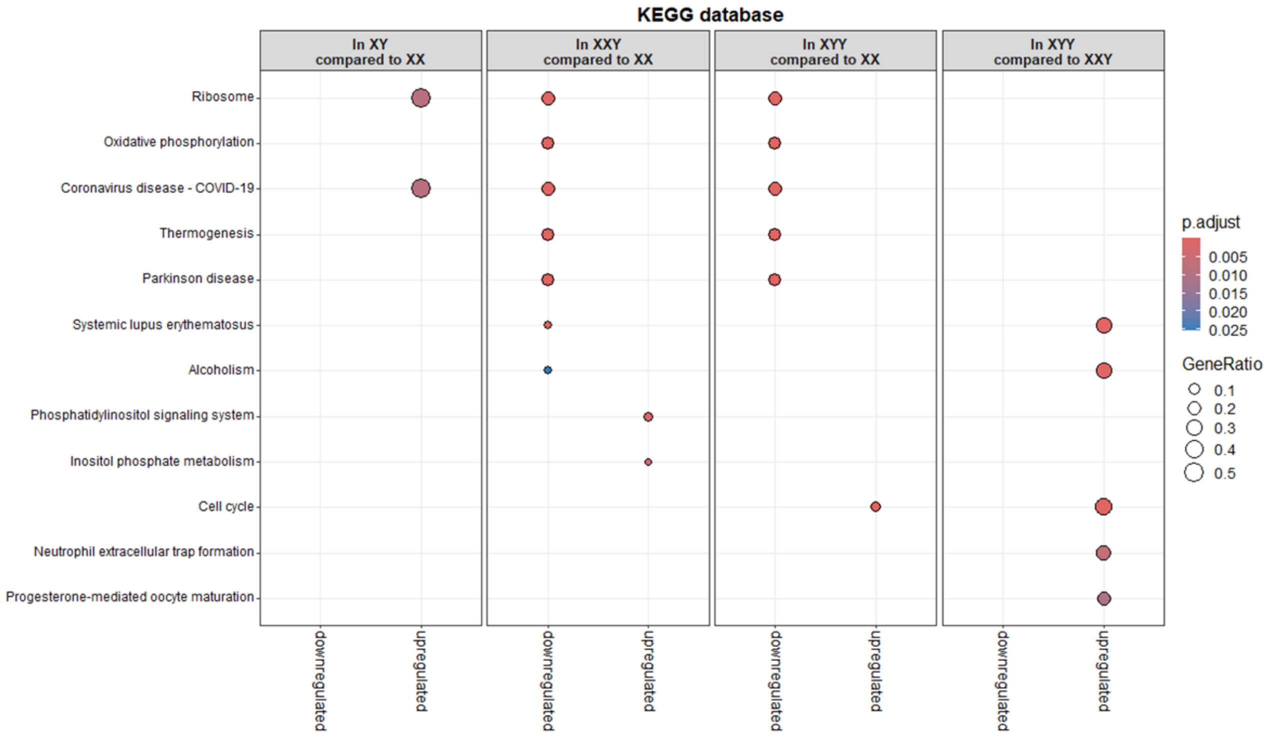

Fig F. Pathway enrichment analysis performed using the differentially expressed genes (regardless if they are protein-coding or not) and the KEGG database.

The plot shows the 5 most significant pathways and shared pathways considering downregulated genes and upregulated genes separately obtained from each pairwise karyotype comparison (when applicable).

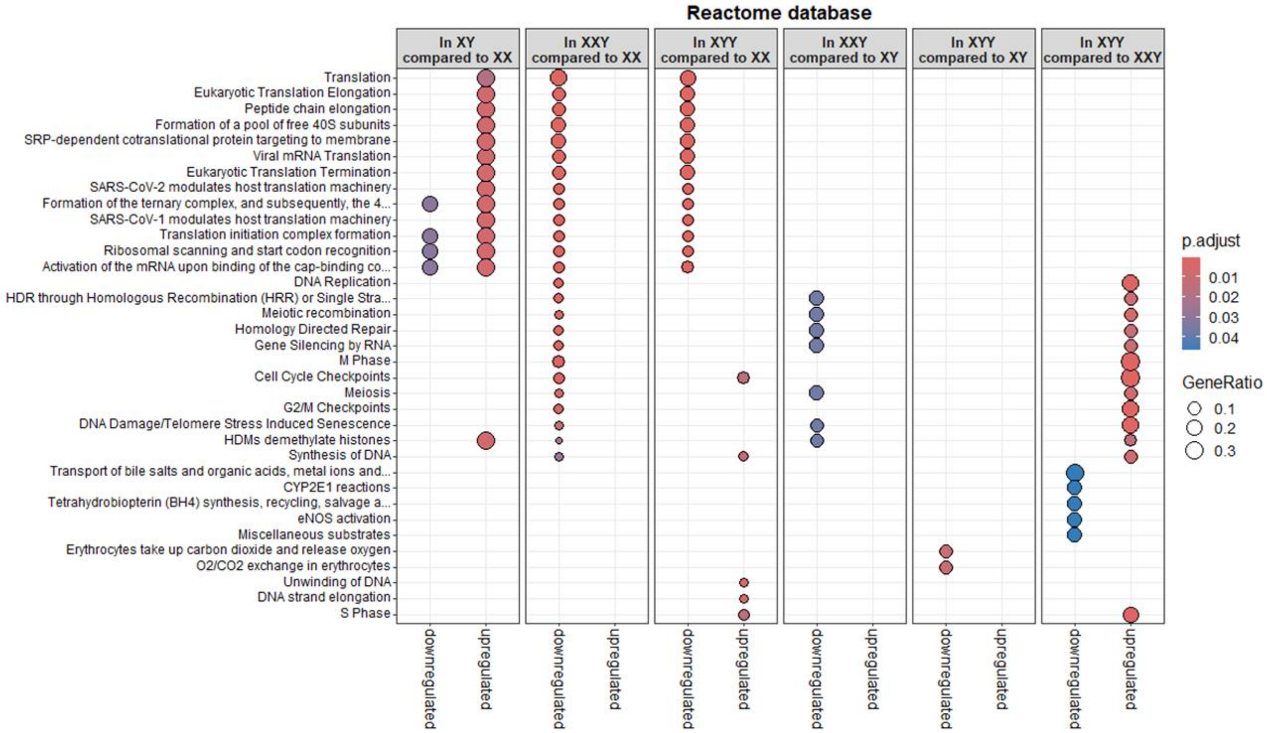

Fig G. Pathway enrichment analysis performed using the differentially expressed genes (regardless if they are protein-coding or not) and the Reactome database.

The plot shows the 5 most significant pathways and shared pathways considering downregulated genes and upregulated genes separately obtained from each pairwise karyotype comparison (when applicable).

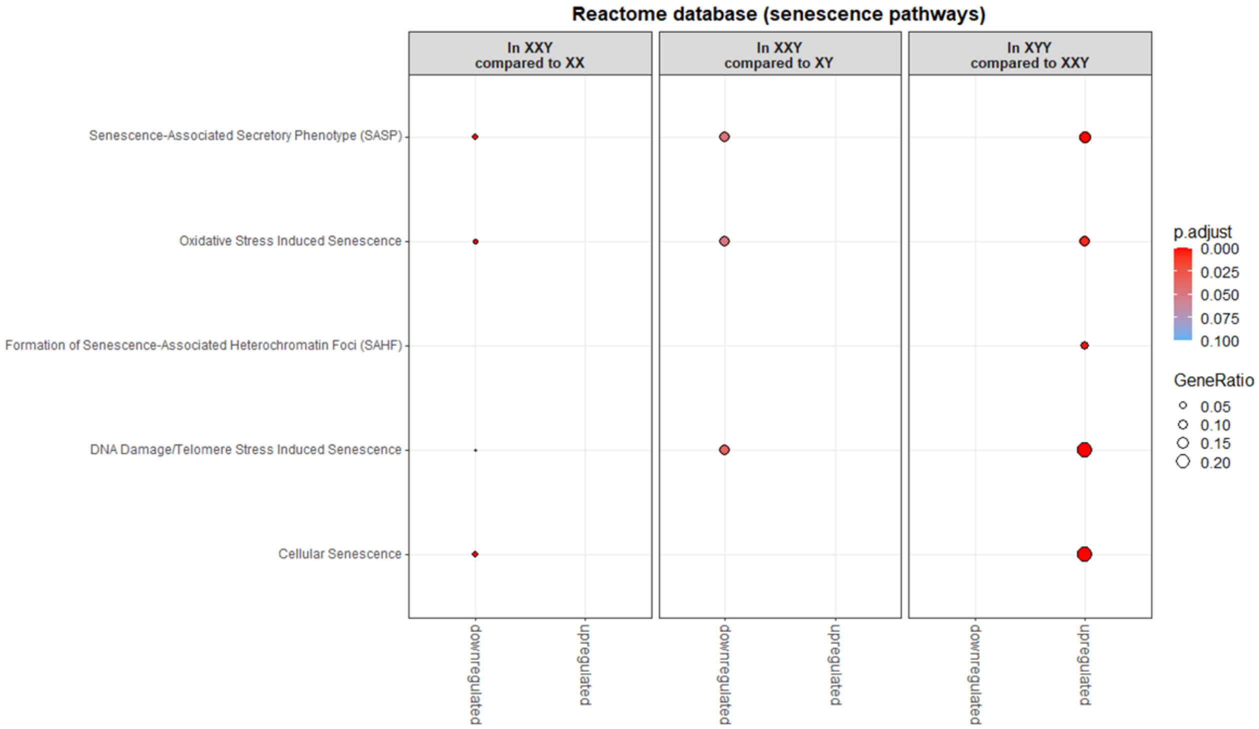

Fig H. Pathway enrichment analysis performed using the differentially expressed genes (regardless if they are protein-coding or not) and focused on the significant terms from the Reactome database including the word “senescence” in their description.

The plot shows the 5 pathways including the word “senescence” in their description if they were significant considering downregulated genes and upregulated genes separately obtained from each pairwise karyotype comparison (when applicable).

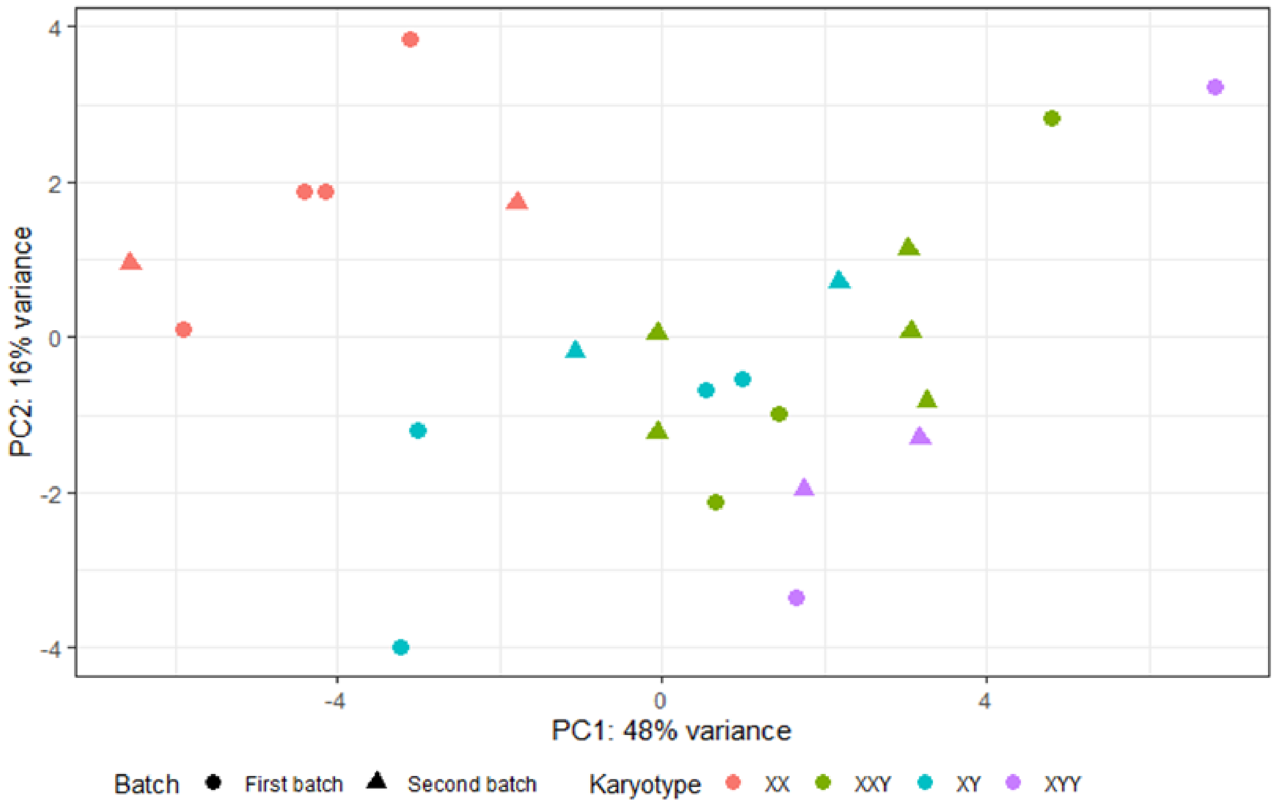

Fig I. Principal component analysis on the 50 TEs explaining the most variance in the gonosome aneuploidy dataset after batch effect was removed.

Each dot represents one individual. Dots are colored according to karyotype (four levels: XX, XY, XXY, XYY) and shaped according to batch (two levels: first batch, second batch). Batch effect was removed before graphical display. PC: Principal Component.

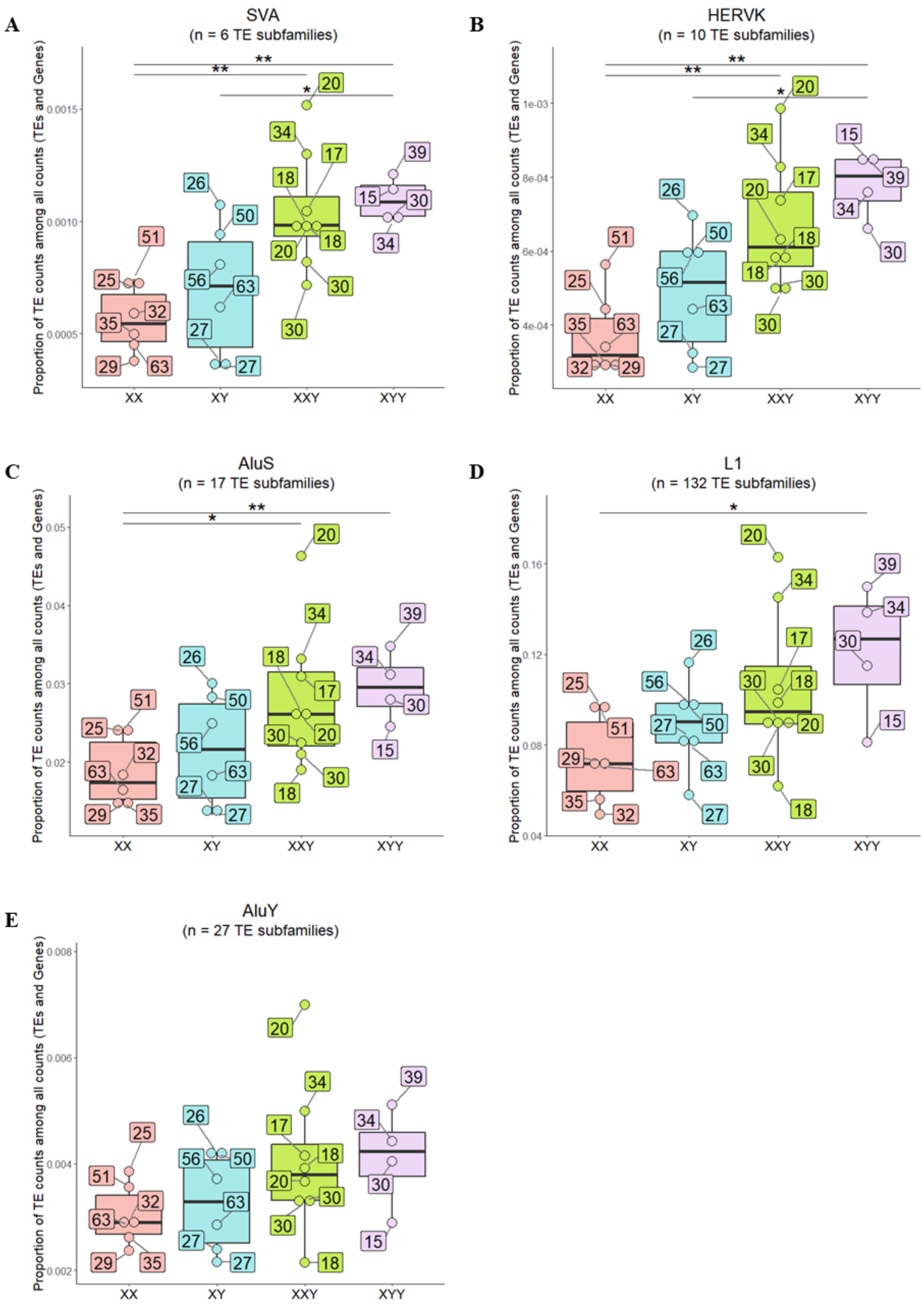
A
SVA
(n = 6 TE subfamilies)
Proportion of TE counts among all counts (TEs and Genes)
0.0015
0.0010
0.0005
XX
XY
XXY
XYY
B
HERVK
(n = 10 TE subfamilies)
Proportion of TE counts among all counts (TEs and Genes)
1e-03
8e-04
6e-04
4e-04
XX
XY
XXY
XYY
C
AluS
(n = 17 TE subfamilies)
Proportion of TE counts among all counts (TEs and Genes)
0.05
0.04
0.03
0.02
XX
XY
XXY
XYY
D
L1
(n = 132 TE subfamilies)
Proportion of TE counts among all counts (TEs and Genes)
0.16
0.12
0.08
0.04
XX
XY
XXY
XYY
E
AluY
(n = 27 TE subfamilies)
Proportion of TE counts among all counts (TEs and Genes)
0.008
0.006
0.004
0.002
XX
XY
XXY
XYY

Fig J. TE expression in the different karyotypes after removing batch effect and considering TE subfamilies belonging to (A) SVA, (B) HERVK, (C) AluS, (D) L1, or (E) AluY elements.

Global TE expression was measured in each karyotype (x-axis) as the proportion of TE read counts among all read counts (TEs and genes) (y-axis). In this calculation, read counts were used after DESeq2 normalization ("normalized counts") to remove any depth sequencing bias. Each dot represents one individual. The age of each individual is in a label box linked to each dot. Batch effect was removed before graphical display. P-values from Wilcoxon test comparing pairwise karyotypes: (*) p-value < 0.05 and (**) p-value < 0.01.

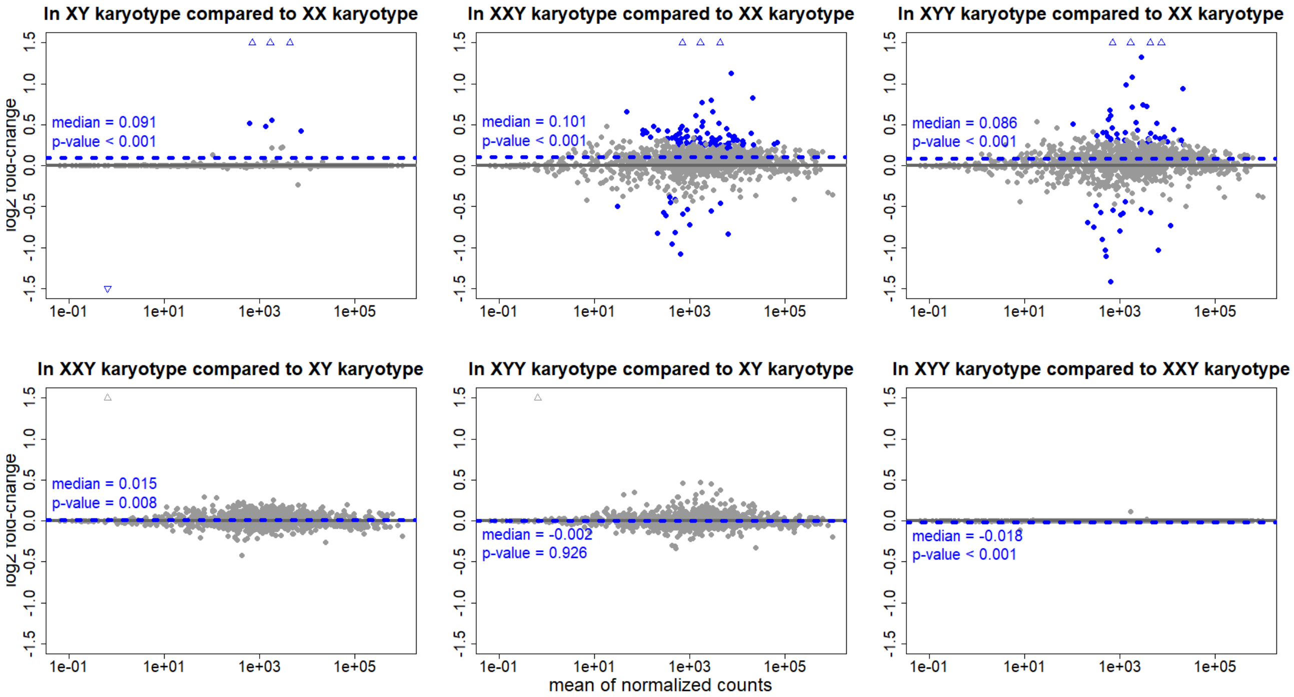

Fig K. MA plots on the 1246 TE subfamilies in each pairwise karyotype comparison after batch adjustment in the gonosome aneuploidy dataset.

Minus-Average plots (MA plots) represent, for each protein-coding gene, the log_2(normalized counts in group 1) – log_2(normalized counts in group 2) on the y-axis and the mean expression (i.e. mean of normalized counts) across all the samples on the x-axis. Each dot or triangle represents one TE subfamily. Blue dots show TE subfamily with an adjusted p-value < 0.05. TE subfamily for which log2 fold-change is greater than 1.5 or less than -1.5 are represented by an empty triangle. The dashed blue line shows the median log2 fold change of the TE subfamilies. The p-value comes from a Wilcoxon test comparing the median log2 fold change of the TE subfamilies to 0 Positive log2 fold-change means that the TE subfamily is upregulated in the first karyotype (e.g. XYY in the “XYY karyotype vs XX karyotype” comparison) compared to the second (e.g. XX in the “XYY karyotype vs XX karyotype” comparison). Negative log2 fold-change means that the TE subfamily is downregulated in the first karyotype (e.g. XYY in the “XYY karyotype vs XX karyotype” comparison) compared to the second (e.g. XX in the “XYY karyotype vs XX karyotype” comparison).

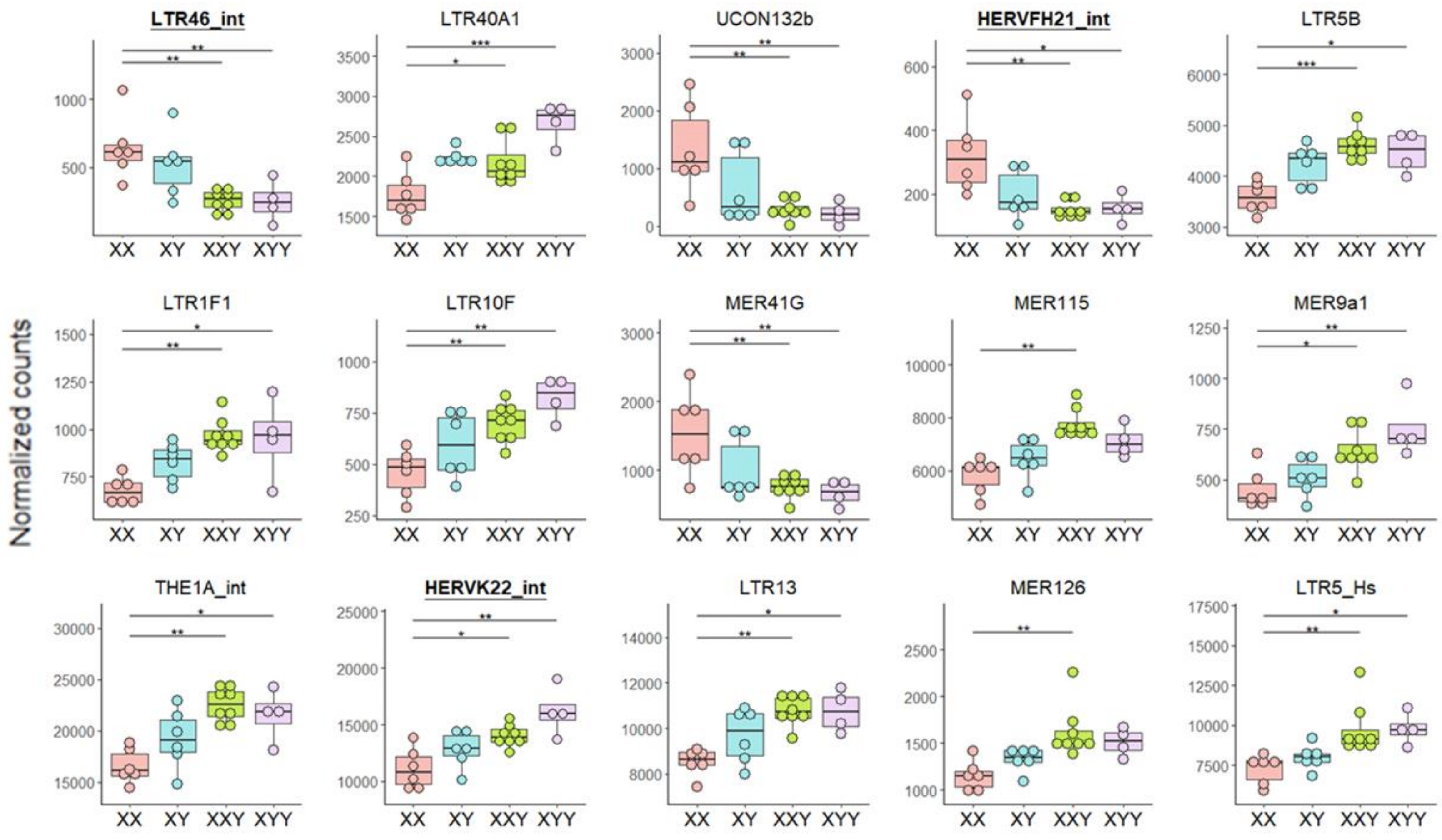

LTR46_int
LTR40A1
UCON132b
HERVFH21_int
LTR5B
LTR1F1
LTR10F
MER41G
MER115
MER9a1
THE1A_int
HERVK22_int
LTR13
MER126
LTR5_Hs
Normalized counts
XX
XY
XXY
XYY

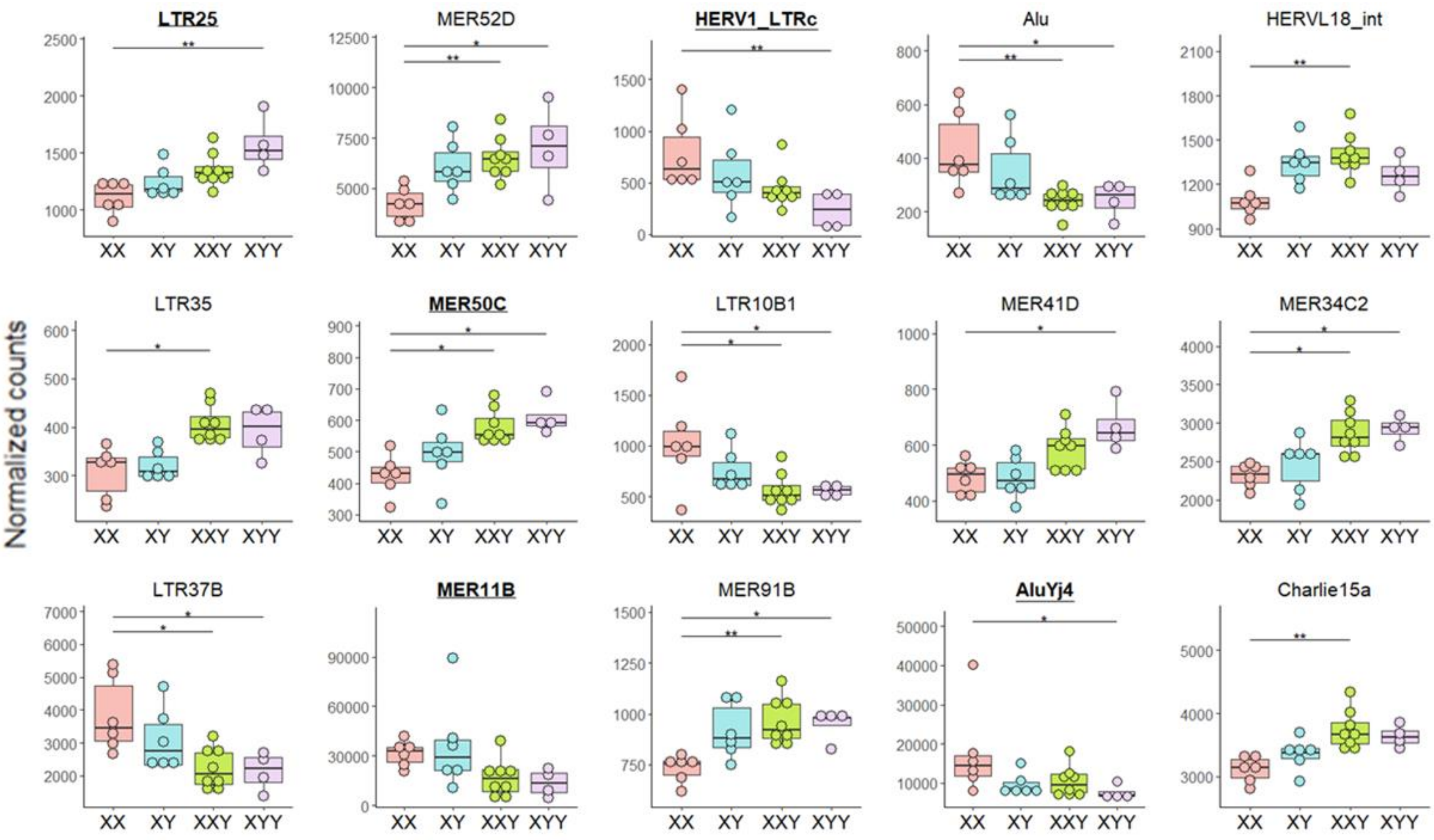
Normalized counts
LTR25
MER52D
HERV1_LTRc
Alu
HERVL18_int
LTR35
MER50C
LTR10B1
MER41D
MER34C2
LTR37B
MER11B
MER91B
AluYj4
Charlie15a
XX XY XXY XYY

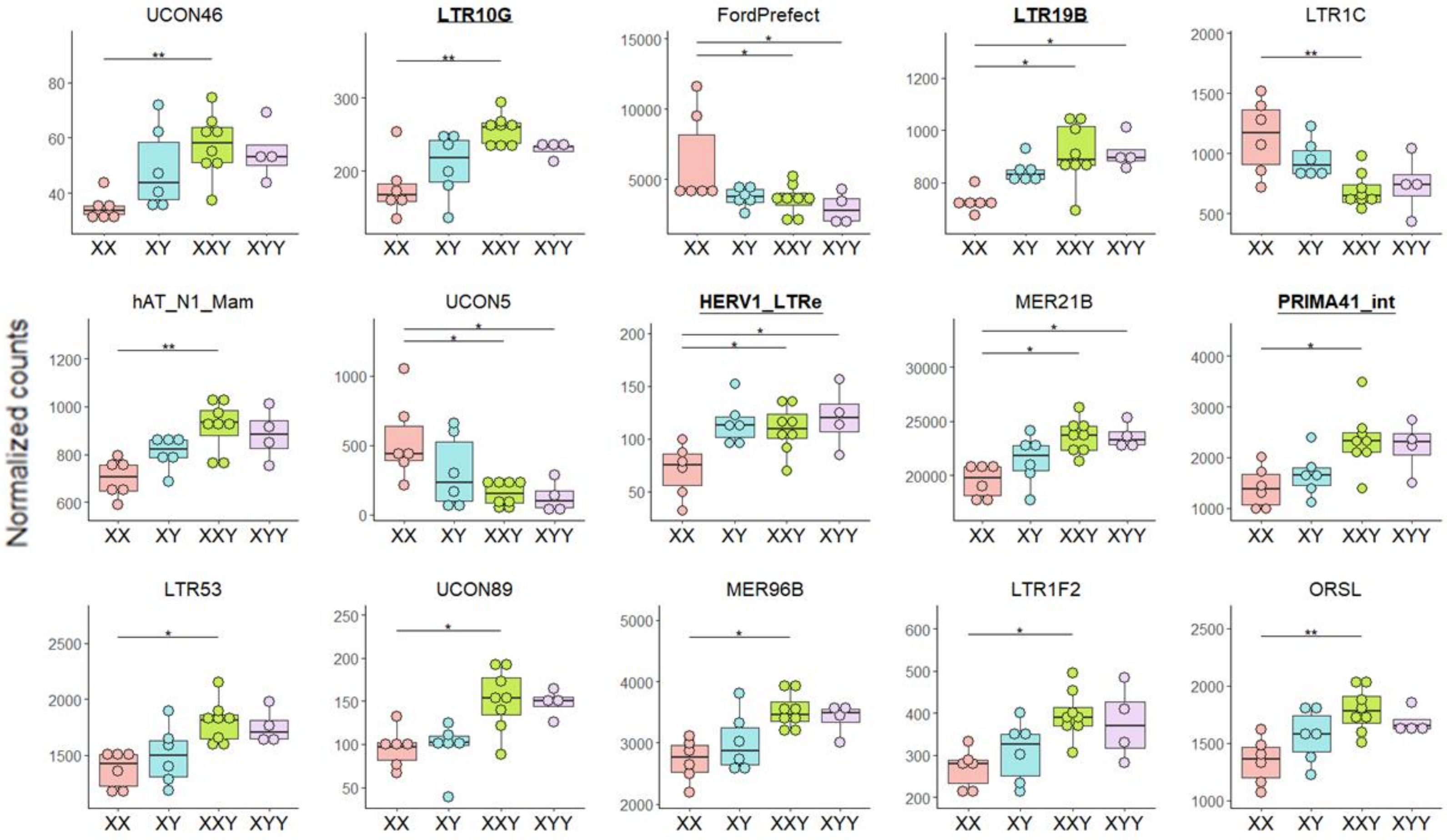

Fig L. Boxplots for the 16th to 60th most significantly differentially expressed TE subfamilies according to karyotype using the likelihood-ratio test, after removing batch effect.

Specific TE subfamily expression was estimated using the number of read counts after DESeq2 normalization ("normalized counts") to remove any depth sequencing bias. Each dot represents one individual. Dots are colored according to karyotype (four levels: XX, XY, XXY, XYY). Batch effect was removed before graphical display. Adjusted p-values from DESeq2: (*) p-value < 0.05, (**) p-value < 0.01, (***) p-value < 0.001. Underlined bold TE subfamilies are enriched in the Y chromosome (binomial test adjusted p-value < 0.05 and number of observed copies located on Y chromosome > expected).

Supplementary Materials

See **Figure 2** for the 15 most significantly differentially expressed TE subfamilies according to karyotype.

See **S5 Data** for number of TE subfamilies upregulated and downregulated per TE family or TE superfamily/order/class in all the pairwise karyotype comparisons.

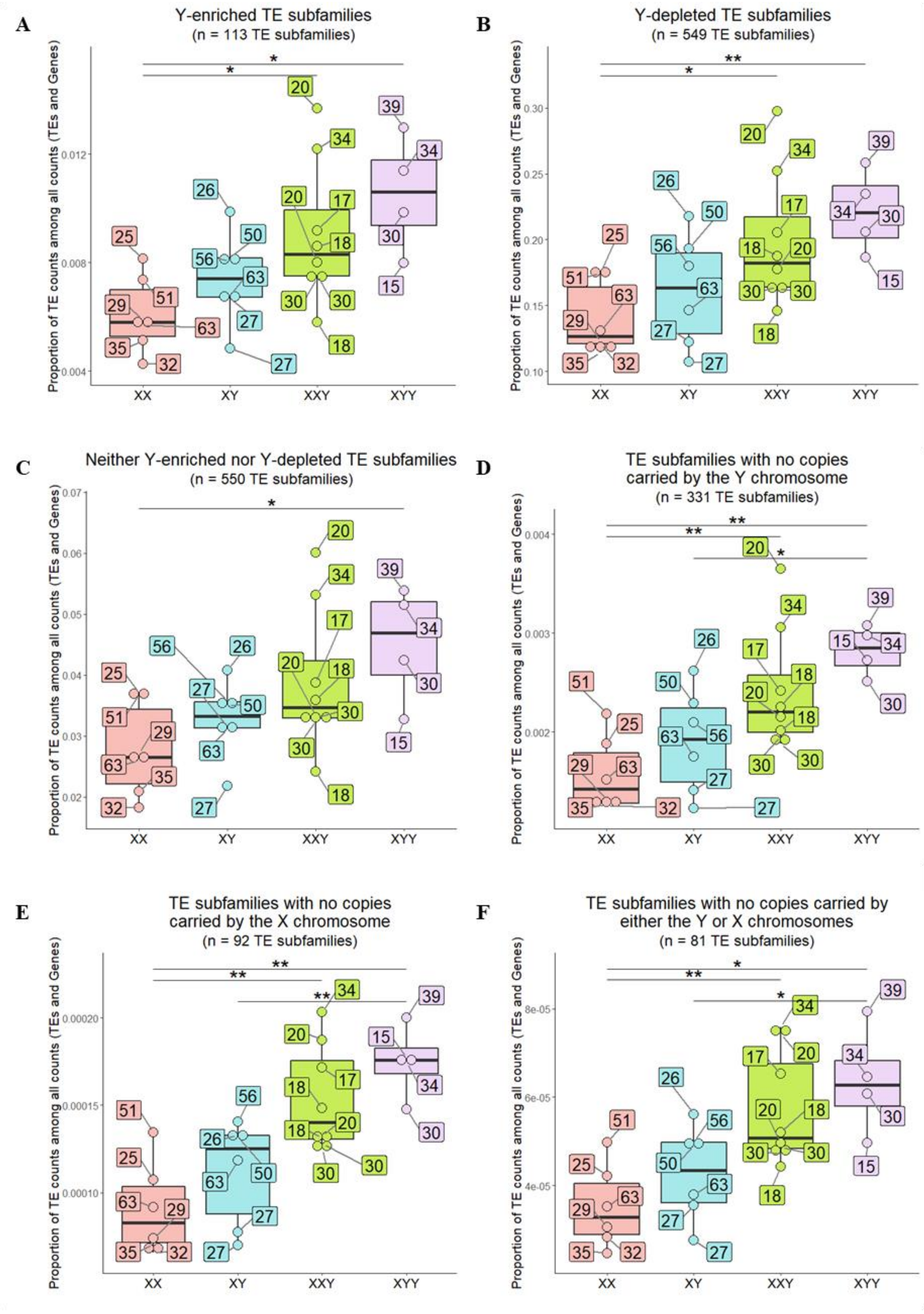
A
Y-enriched TE subfamilies
(n = 113 TE subfamilies)
B
Y-depleted TE subfamilies
(n = 549 TE subfamilies)
C
Neither Y-enriched nor Y-depleted TE subfamilies
(n = 550 TE subfamilies)
D
TE subfamilies with no copies carried by the Y chromosome
(n = 331 TE subfamilies)
E
TE subfamilies with no copies carried by the X chromosome
(n = 92 TE subfamilies)
F
TE subfamilies with no copies carried by either the Y or X chromosomes
(n = 81 TE subfamilies)
Proportion of TE counts among all counts (TEs and Genes)
XX
XY
XXY
XYY

Fig M. TE expression in the different karyotypes after removing batch effect and considering all TE subfamilies which were (A) Y-enriched TE subfamilies, (B) Y-depleted TE subfamilies, (C) neither Y-enriched nor Y-depleted TE subfamilies, (D) TE subfamilies with no copies carried by the Y chromosome, (E) TE subfamilies with no copies carried by the X chromosome, or (F) TE subfamilies with no copies carried by either the Y or X chromosomes.

Global TE expression was measured in each karyotype (x-axis) as the proportion of TE read counts among all read counts (TEs and genes) (y-axis). In this calculation, read counts were used after DESeq2 normalization ("normalized counts") to remove any depth sequencing bias. Each dot represents one individual. The age of each individual is in a label box linked to each dot. Batch effect was removed before graphical display. P-values from Wilcoxon test comparing pairwise karyotypes: (*) p-value < 0.05 and (**) p-value < 0.01.

See **S7 Data** to know which TE subfamilies are Y-enriched (binomial test adjusted p-value < 0.05 and number of observed copies located on Y chromosome > expected)), Y-depleted (binomial test adjusted p-value < 0.05 and number of observed copies located on Y chromosome < expected) and to know the number of TE copy carried by the X and Y chromosomes according to the human reference sequence file of TE insertions.

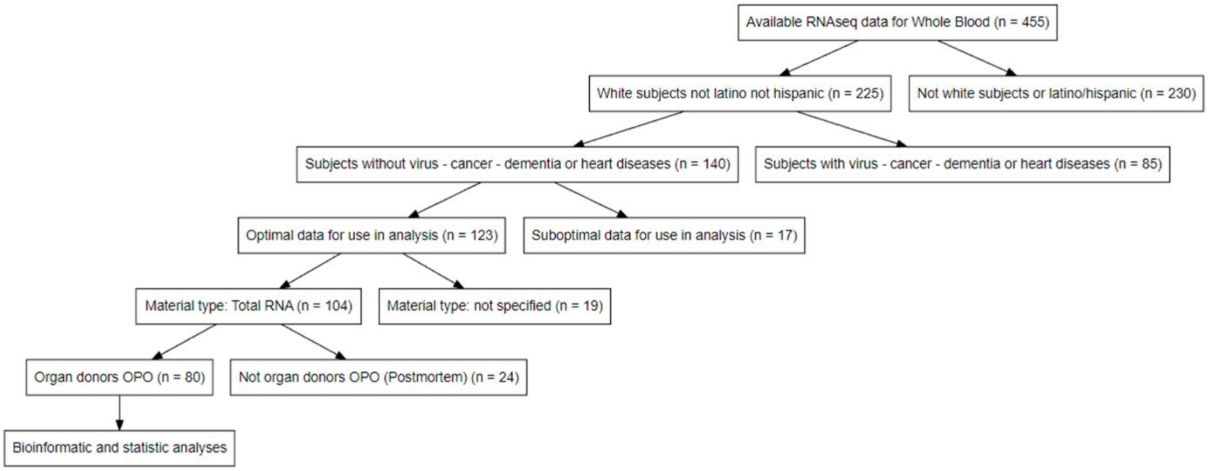

Fig N. Flowchart showing the selection criteria used to constitute the filtered GTEx dataset (no disease group).

OPO: Organ Procurement Organization.

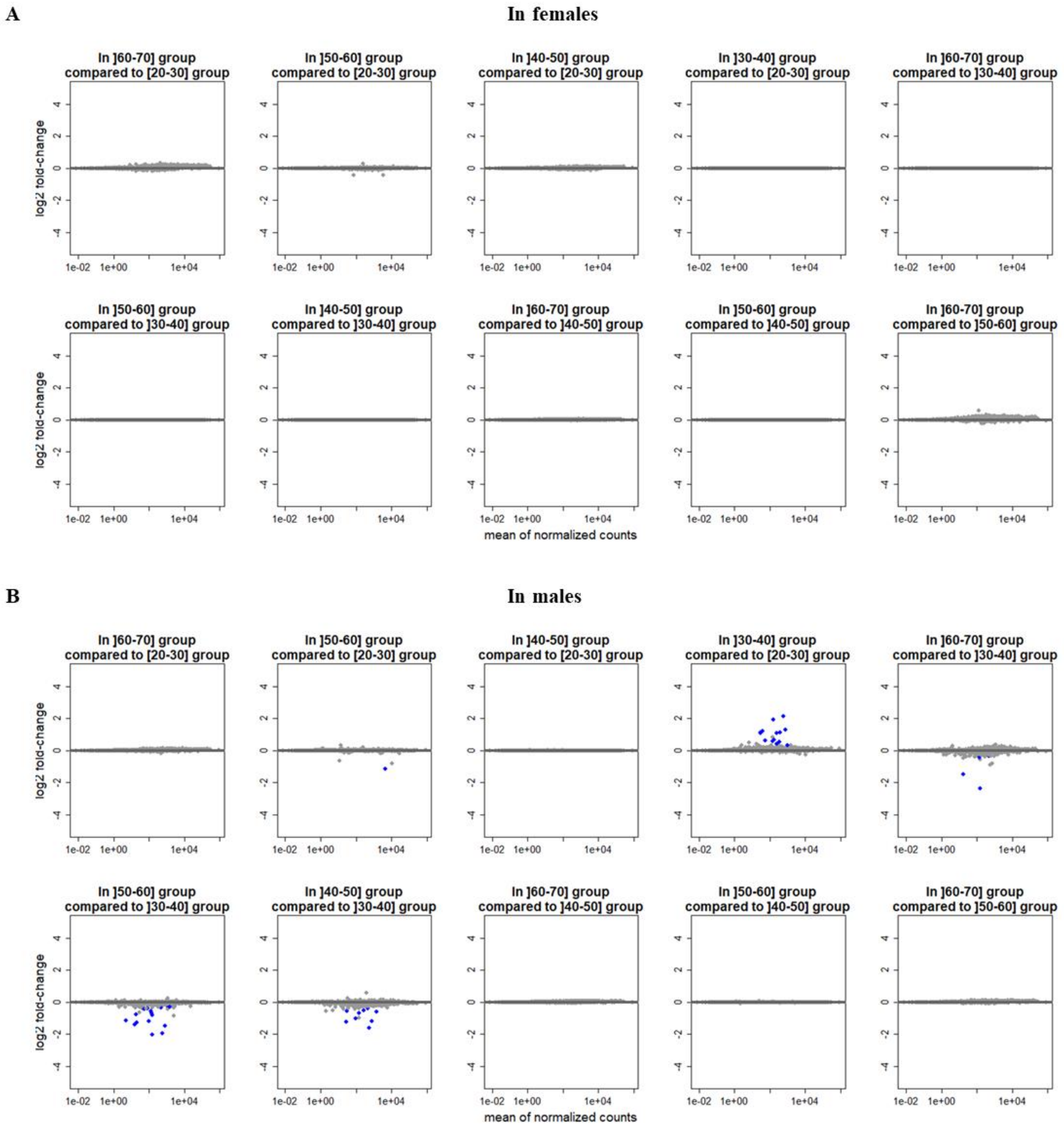
A
In females
In]60-70] group compared to [20-30] group
In]50-60] group compared to [20-30] group
In]40-50] group compared to [20-30] group
In]30-40] group compared to [20-30] group
In]60-70] group compared to]30-40] group
In]50-60] group compared to]30-40] group
In]40-50] group compared to]30-40] group
In]60-70] group compared to]40-50] group
In]50-60] group compared to]40-50] group
In]60-70] group compared to]50-60] group
log2 fold-change
mean of normalized counts
B
In males
In]60-70] group compared to [20-30] group
In]50-60] group compared to [20-30] group
In]40-50] group compared to [20-30] group
In]30-40] group compared to [20-30] group
In]60-70] group compared to]30-40] group
In]50-60] group compared to]30-40] group
In]40-50] group compared to]30-40] group
In]60-70] group compared to]40-50] group
In]50-60] group compared to]40-50] group
In]60-70] group compared to]50-60] group
log2 fold-change
mean of normalized counts

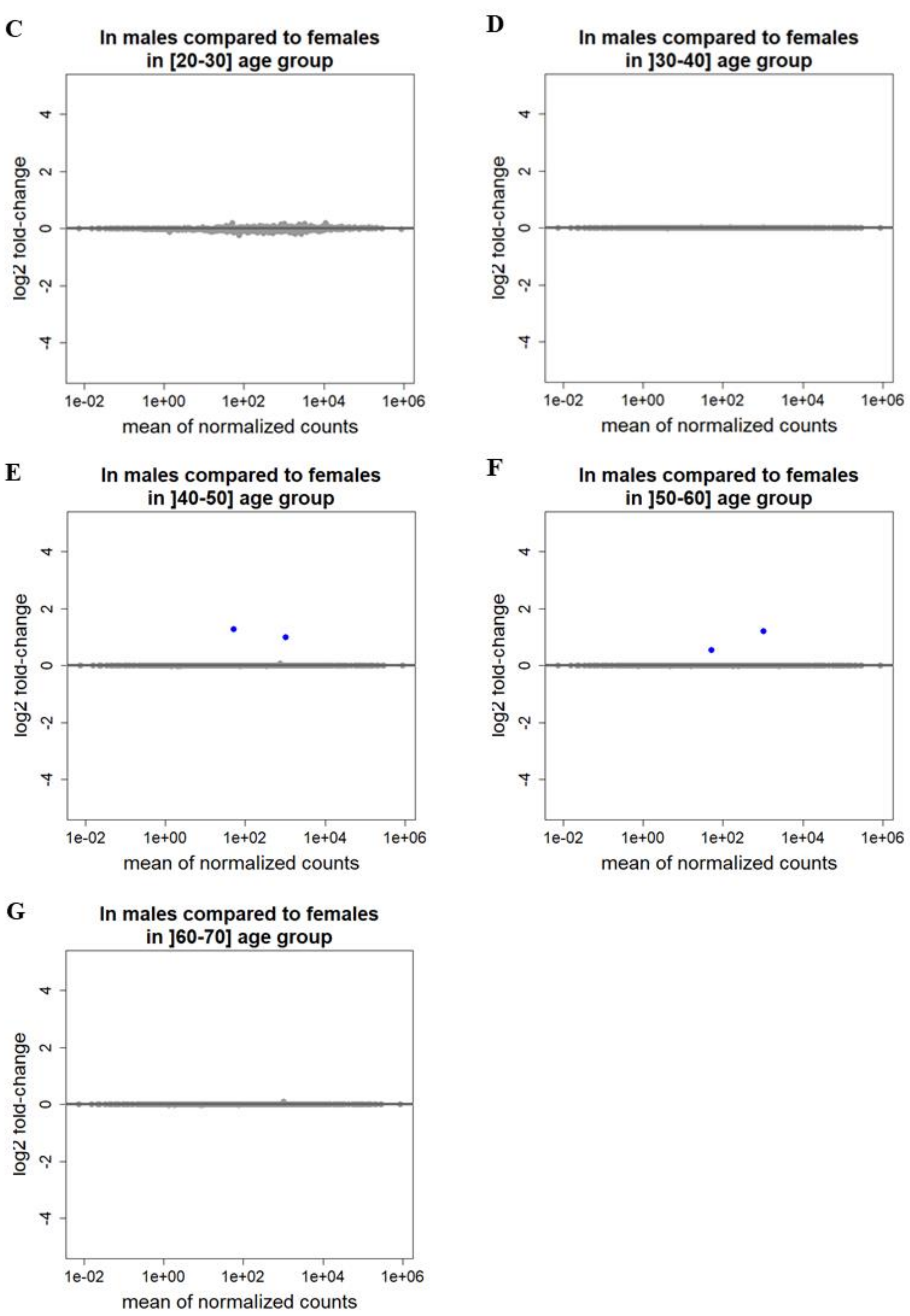
C
In males compared to females
in [20-30] age group
D
In males compared to females
in]30-40] age group
E
In males compared to females
in]40-50] age group
F
In males compared to females
in]50-60] age group
G
In males compared to females
in]60-70] age group
log2 fold-change
-4
-2
0
2
4
mean of normalized counts
1e-02
1e+00
1e+02
1e+04
1e+06

Fig O. MA plots on the 1246 TE subfamilies using the filtered GTEx dataset (no disease group): (A) pairwise comparisons of age group in females, (B) pairwise comparisons of age group in males, (C) comparison between females and males in age group 20-30 years old, (D) comparison between females and males in age group 30-40 years old, (E) comparison between females and males in age group 40-50 years old, (F) comparison between females and males in age group 50-60 years old, and (G) comparison between females and males in age group 60-70 years old.

Minus-Average plots (MA plots) represent, for each protein-coding gene, the $\log_2$(normalized counts in group 1) – $\log_2$(normalized counts in group 2) on the y-axis and the mean expression (i.e. mean of normalized counts) across all the samples on the x-axis. Each dot represents one TE subfamily. Blue dots show TE subfamilies with an adjusted p-value < 0.05. Positive log2 fold-change means that the TE subfamily is upregulated in the first group (e.g. Males in the “Males vs Females” comparison) compared to the second (e.g. Females in the “Males vs Females” comparison). Negative log2 fold-change means that the TE subfamily is downregulated in the first group (e.g. Males in the “Males vs Females” comparison) compared to the second (e.g. Females in the “Males vs Females” comparison).

Supplementary Materials

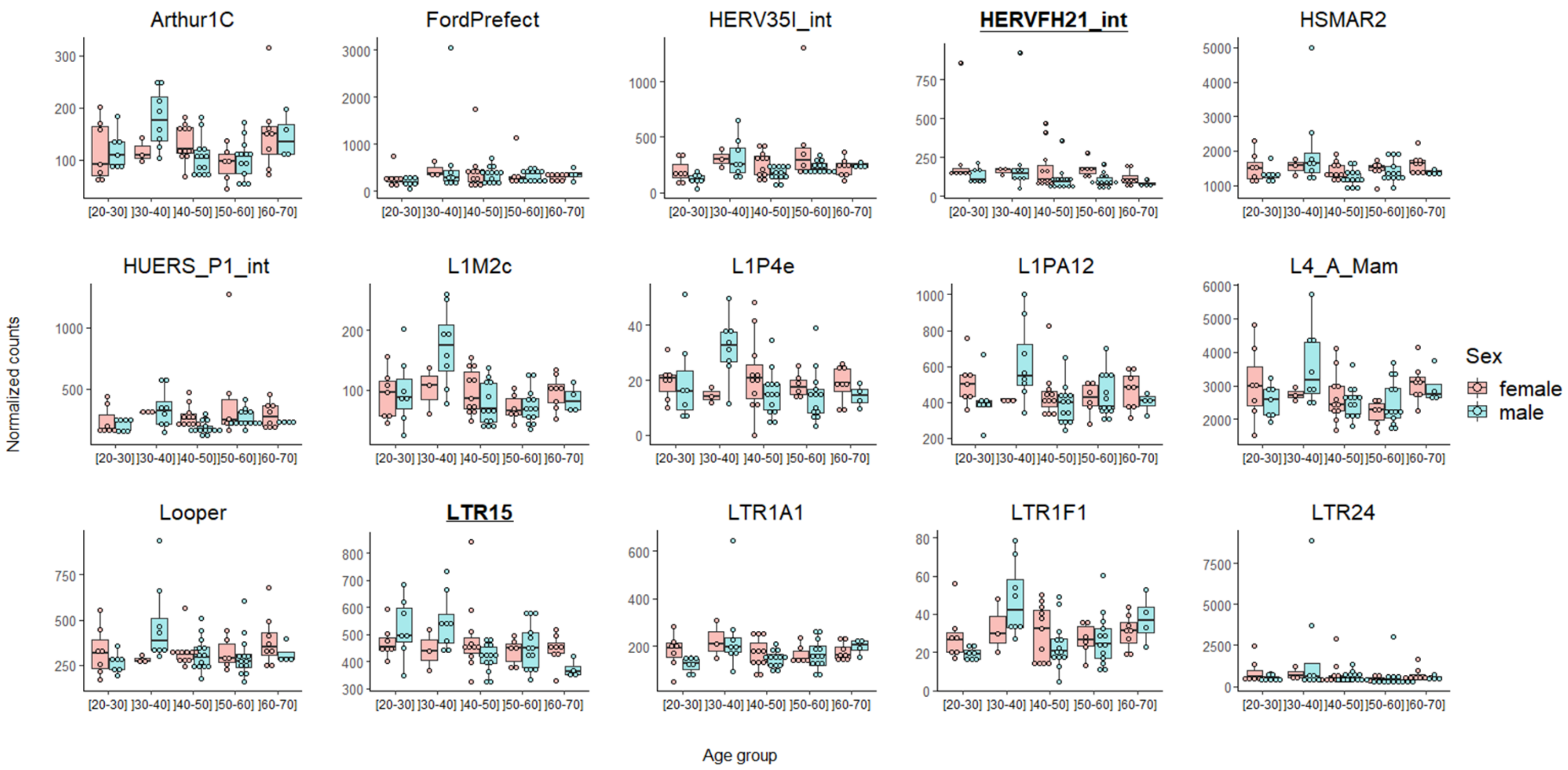

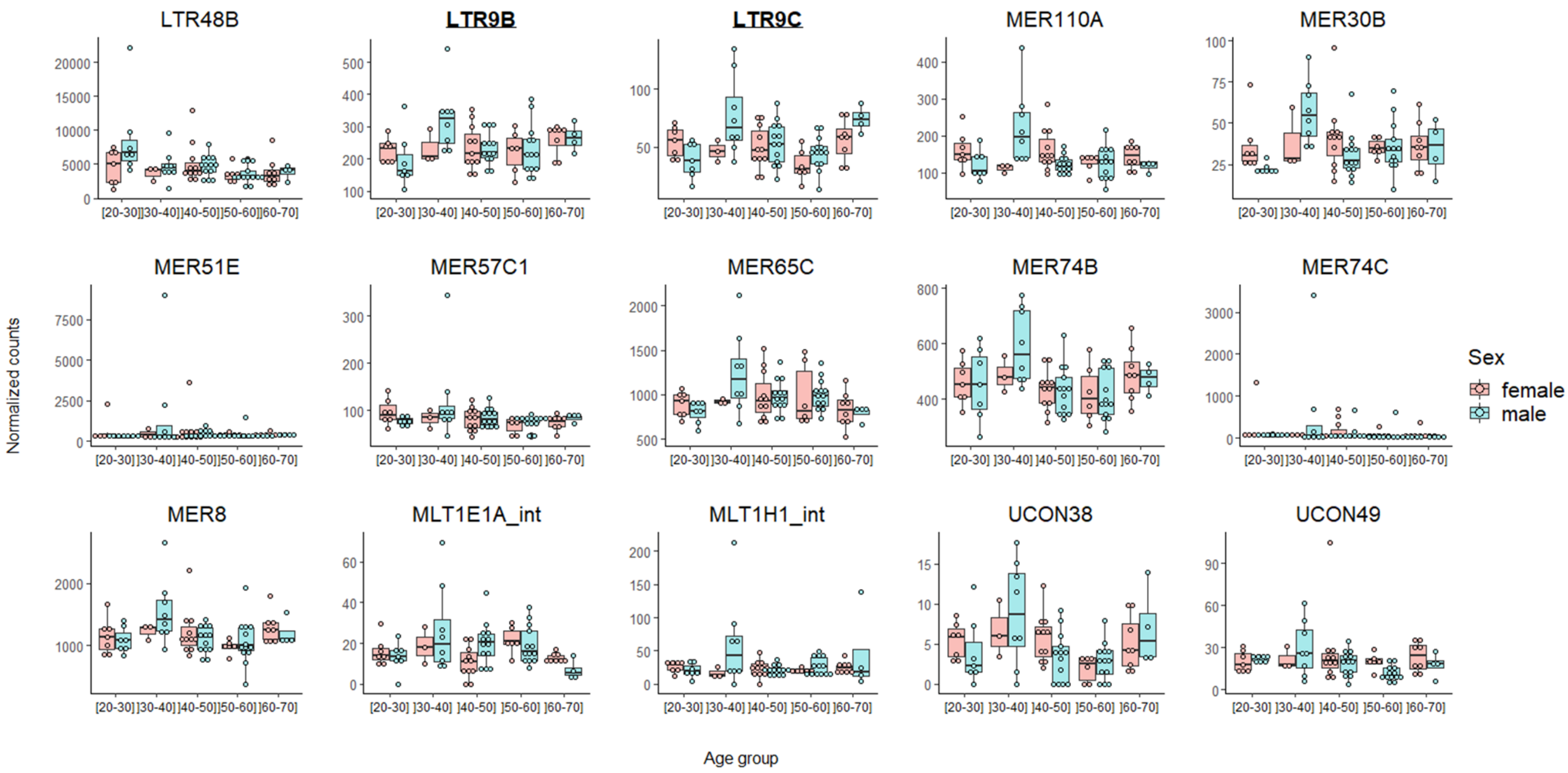
LTR48B
LTR9B
LTR9C
MER110A
MER30B
MER51E
MER57C1
MER65C
MER74B
MER74C
MER8
MLT1E1A_int
MLT1H1_int
UCON38
UCON49
Normalized counts
Age group
[20-30]
]30-40]
]40-50]
]50-60]
]60-70]
Sex
female
male

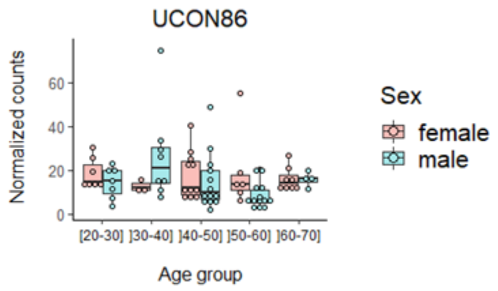

Fig P. Boxplots of normalized counts according to sex and age group, for TE subfamilies that are significantly differentially expressed between two age groups in males or females in the filtered GTEx dataset (no disease group).

Specific TE subfamily expression was estimated using the number of read counts after DESeq2 normalization ("normalized counts") to remove any depth sequencing bias. Each dot represents one individual. Dots are colored according to sex. Sex variable has two levels: female, male. Age group has five levels: [20-30],]30-40],]40-50],]50-60], and]60-70]. Underlined bold TE subfamilies are enriched in the Y chromosome (binomial test adjusted p-value < 0.05 and number of observed copies located on Y chromosome > expected).

For more details on the group comparisons in which TE subfamilies were significantly differentially expressed, see **Table I**.

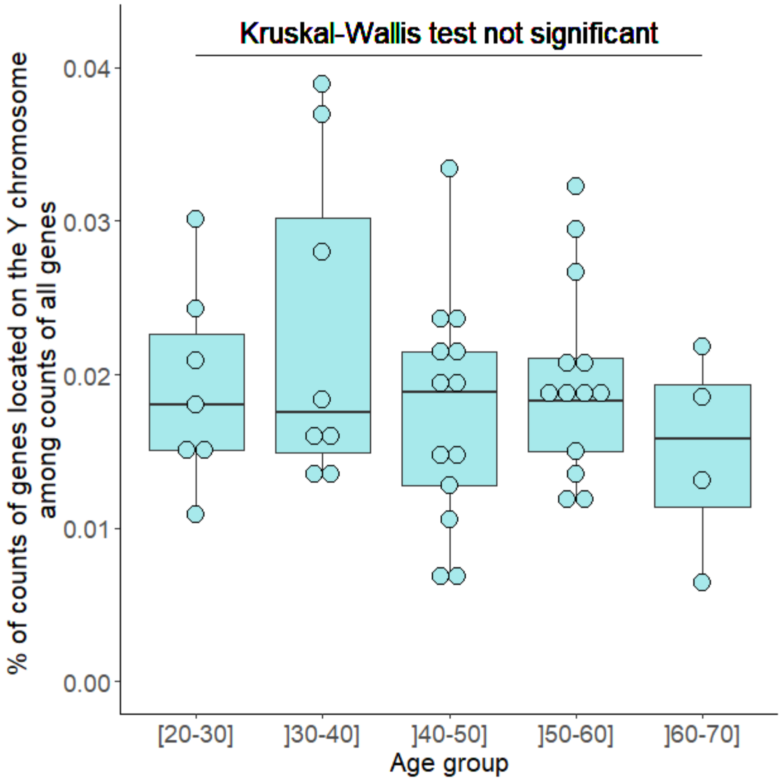

Fig Q. Expression of genes located on the Y chromosome in males in the filtered GTEx dataset (no disease group) according to age group.

A Kruskal-Wallis test comparing the percentage of counts of genes located on the Y chromosome among counts of all genes across age groups was used and was not significant in females (p-value = 0.823).

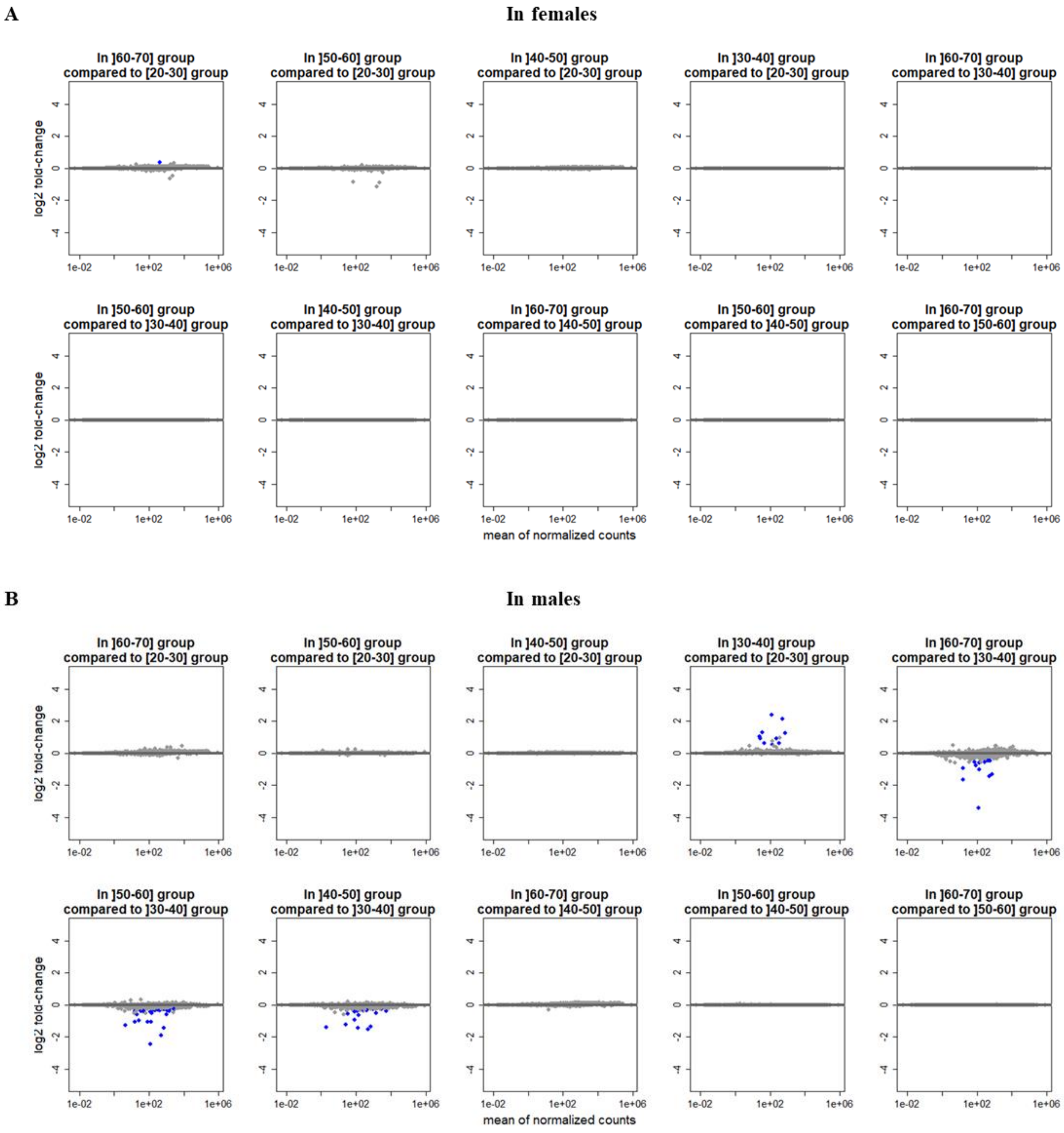
A
In females
In]60-70] group compared to [20-30] group
In]50-60] group compared to [20-30] group
In]40-50] group compared to [20-30] group
In]30-40] group compared to [20-30] group
In]60-70] group compared to]30-40] group
In]50-60] group compared to]30-40] group
In]40-50] group compared to]30-40] group
In]60-70] group compared to]40-50] group
In]50-60] group compared to]40-50] group
In]60-70] group compared to]50-60] group
log2 fold-change
mean of normalized counts
1e-02
1e+02
1e+06
B
In males
In]60-70] group compared to [20-30] group
In]50-60] group compared to [20-30] group
In]40-50] group compared to [20-30] group
In]30-40] group compared to [20-30] group
In]60-70] group compared to]30-40] group
In]50-60] group compared to]30-40] group
In]40-50] group compared to]30-40] group
In]60-70] group compared to]40-50] group
In]50-60] group compared to]40-50] group
In]60-70] group compared to]50-60] group
log2 fold-change
mean of normalized counts

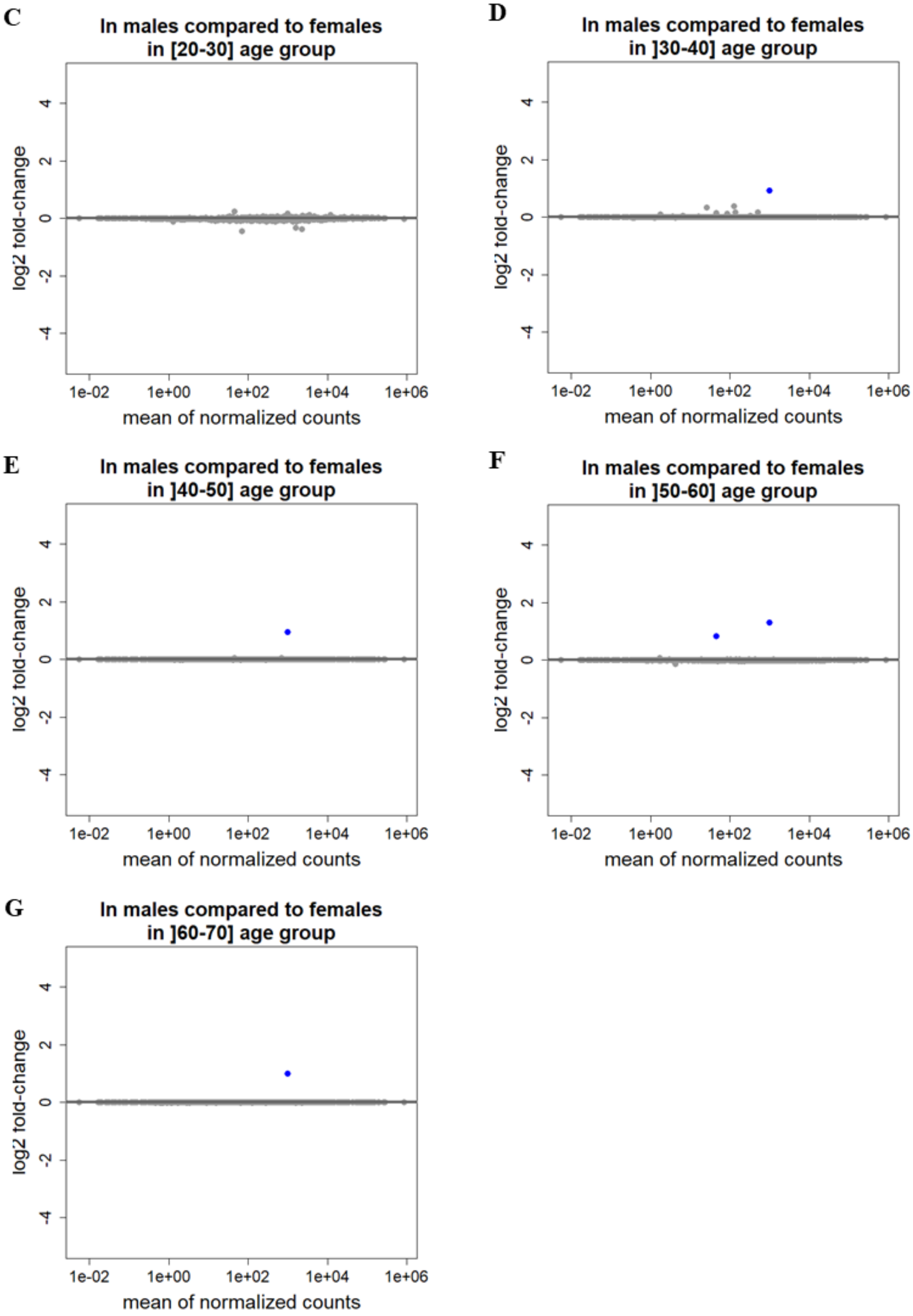

Fig R. MA plots on the 1246 TE subfamilies using the filtered GTEx dataset (group including subjects with current and antecedents of cancer and cardiovascular diseases): (A) pairwise comparisons of age group in

females, (B) pairwise comparisons of age group in males, (C) comparison between females and males in age group 20-30 years old, (D) comparison between females and males in age group 30-40 years old, (E) comparison between females and males in age group 40-50 years old, (F) comparison between females and males in age group 50-60 years old, and (G) comparison between females and males in age group 60-70 years old.

Minus-Average plots (MA plots) represent, for each protein-coding gene, the log_2(normalized counts in group 1) – log_2(normalized counts in group 2) on the y-axis and the mean expression (i.e. mean of normalized counts) across all the samples on the x-axis. Each dot represents one TE subfamily. Blue dots show TE subfamilies with an adjusted p-value < 0.05. Positive log2 fold-change means that the TE subfamily is upregulated in the first group (e.g. Males in the "Males vs Females" comparison) compared to the second (e.g. Females in the "Males vs Females" comparison). Negative log2 fold-change means that the TE subfamily is downregulated in the first group (e.g. Males in the "Males vs Females" comparison) compared to the second (e.g. Females in the "Males vs Females" comparison).

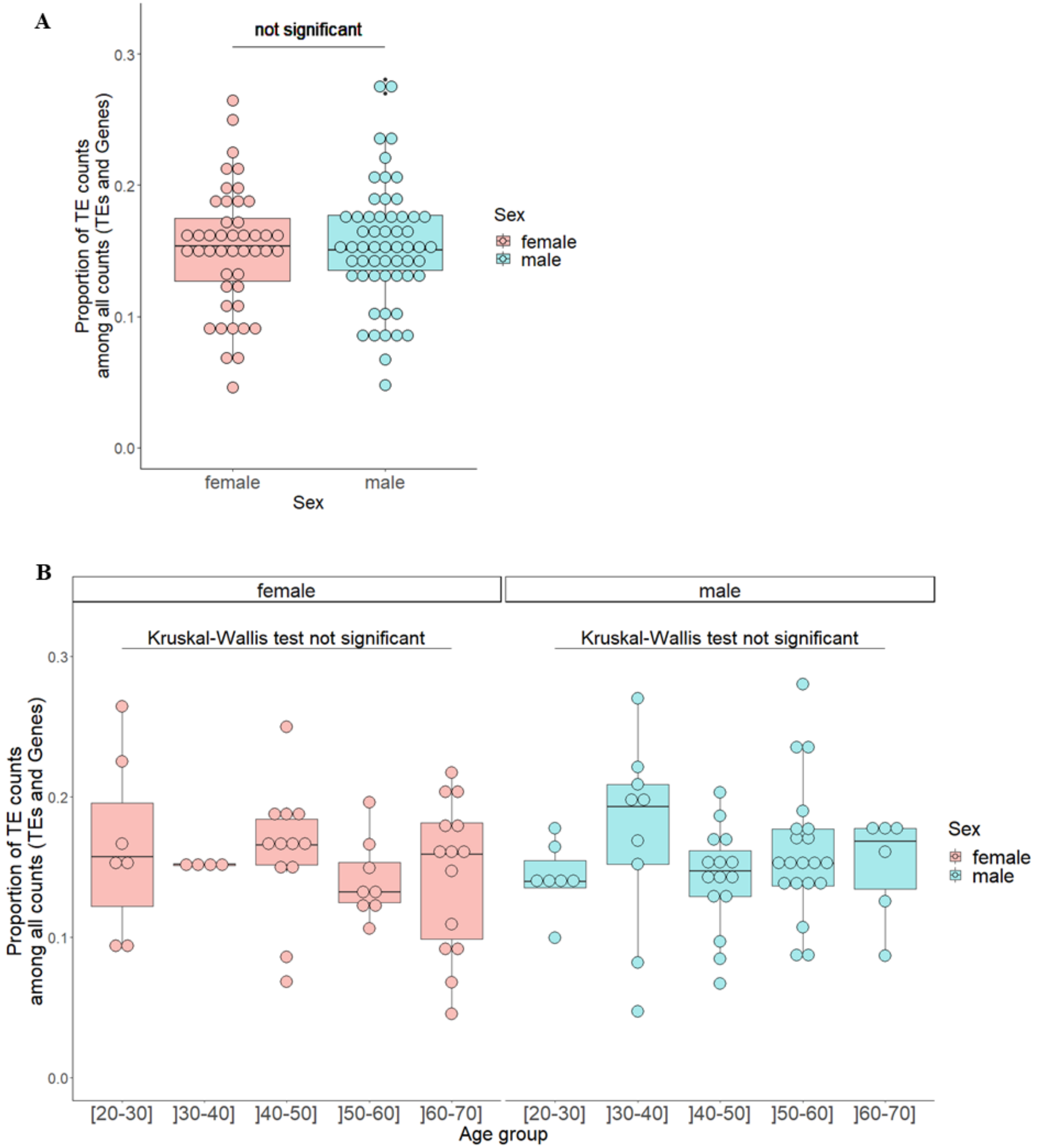

Fig S. TE expression in the filtered GTEx dataset (in the group including subjects with current and antecedents of cancer and cardiovascular diseases).

Global TE expression was measured in each karyotype (x-axis) as the proportion of TE read counts among all read counts (TEs and genes) (y-axis). In this calculation, read counts were used after DESeq2 normalization (“normalized counts”) to remove any depth sequencing bias. Each dot represents one individual. Dots are

colored according to sex. Sex variable has two levels: female, male. Age group has five levels: [20-30],]30-40],]40-50],]50-60], and]60-70].

(**A**) Proportion of TE read counts among all read counts was modelized using a linear model with sex and age group as independent variables. From this model, the p-value testing the effect of sex on proportion of TE counts among all counts adjusted on age group was not significant.

(**B**) A Kruskal-Wallis test comparing proportion of TE read counts among all read counts across age groups was used in females and then on males. This test was not significant in females as well as in males.

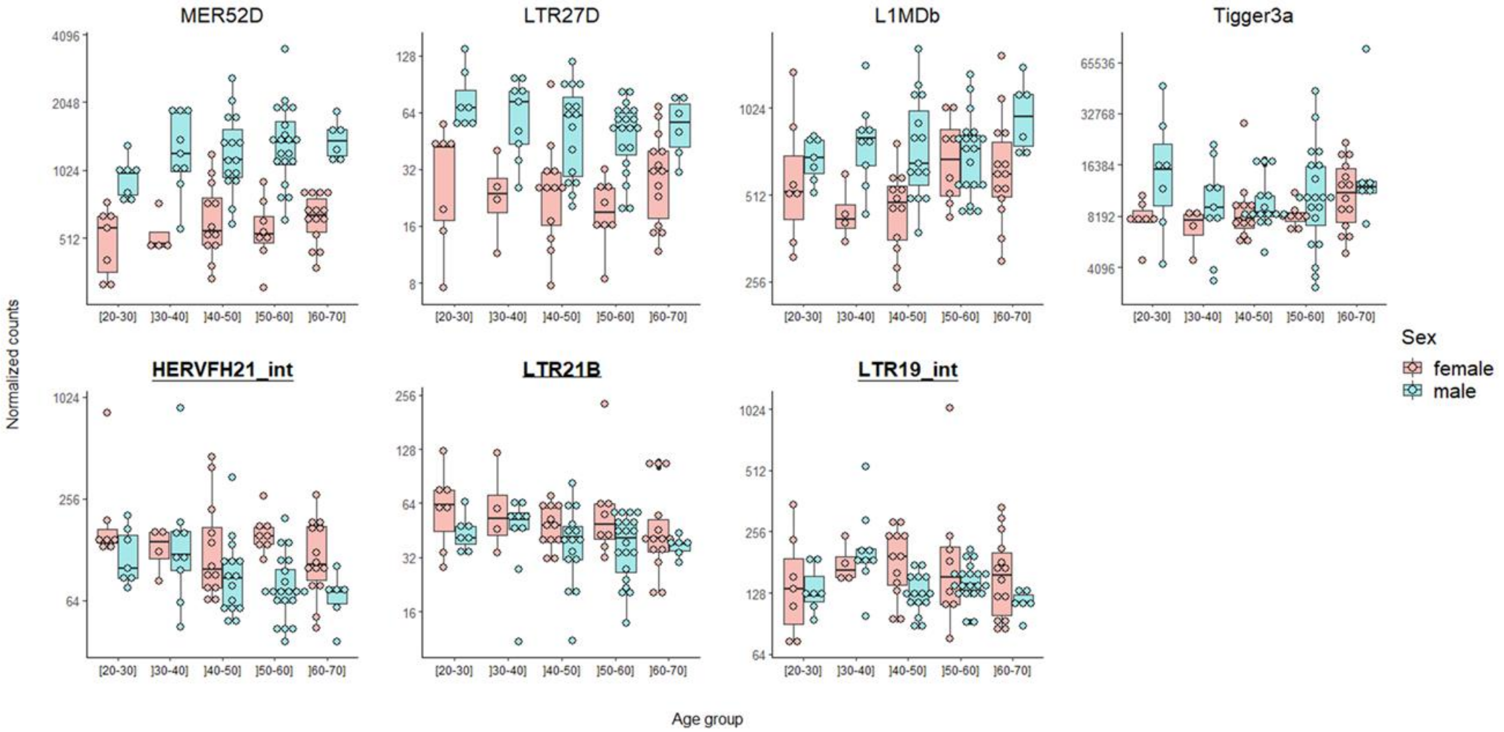

Fig T. Boxplots of normalized counts according to sex and age group, for the TE subfamilies that are significantly differentially expressed between males and females in at least one age group or adjusted on age group in the filtered GTEx dataset (in the group including subjects with current and antecedents of cancer and cardiovascular diseases).

Specific TE subfamily expression was estimated using the number of read counts after DESeq2 normalization ("normalized counts") to remove any depth sequencing bias. Each dot represents one individual. Dots are colored according to sex. Sex variable has two levels: female, male. Age group has five levels: [20-30],]30-40],]40-50],]50-60], and]60-70]. Y-axis is on a log2 scale.

All of these subfamilies had at least one copy on the Y chromosome and one copy on the X chromosome. Underlined bold TE subfamilies are enriched in the Y chromosome (binomial test adjusted p-value < 0.05 and number of observed copies located on Y chromosome > expected).

For more details on the group comparisons in which TE subfamilies were significantly differentially expressed, see **Table L**.

A

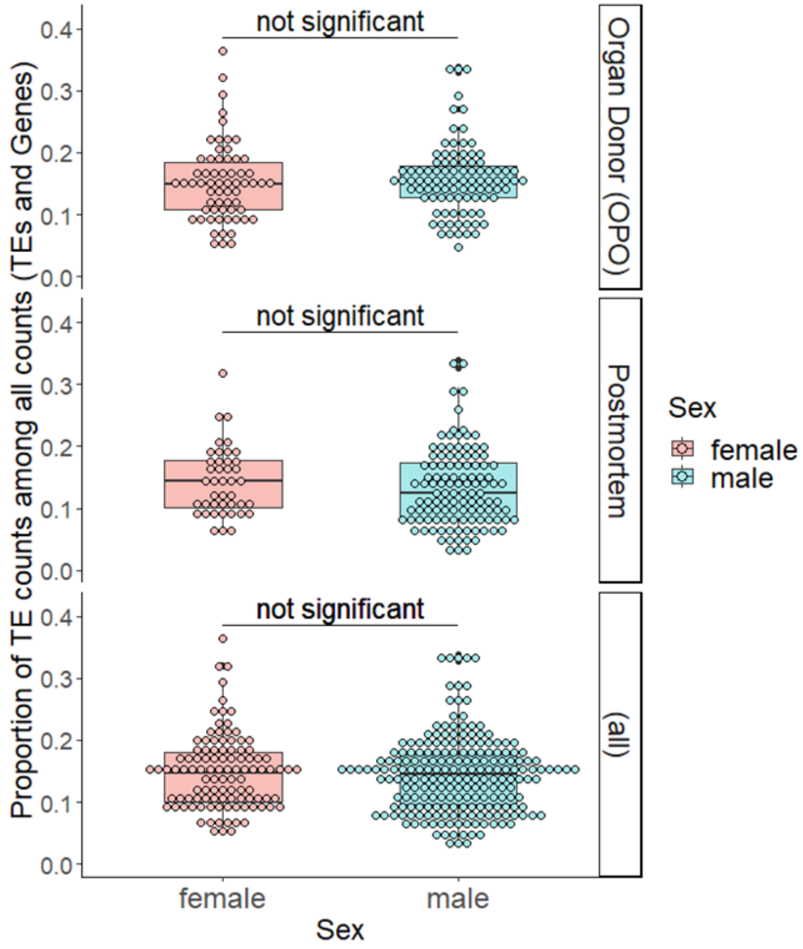
not significant
not significant
not significant
Organ Donor (OPO)
Postmortem
(all)
Proportion of TE counts among all counts (TEs and Genes)
0.0
0.1
0.2
0.3
0.4
female
male
Sex
Sex
female
male

B

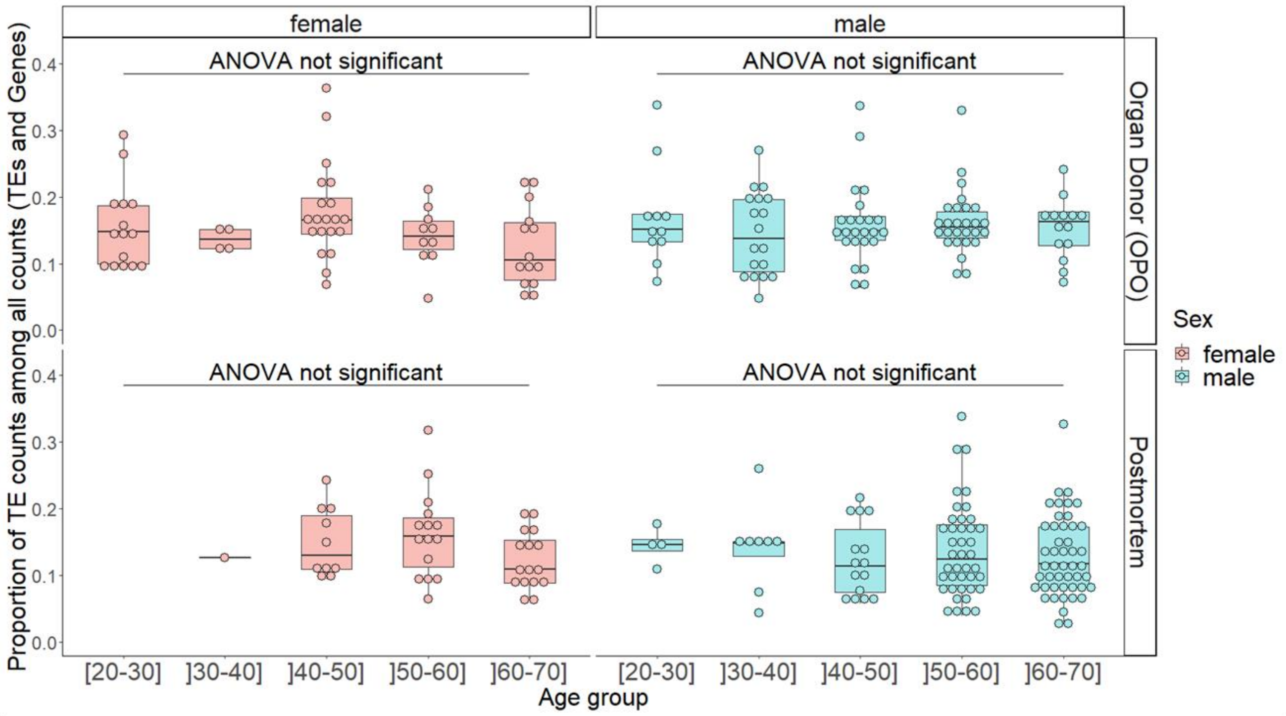

Fig U. TE expression in the large GTEx dataset according to "sex" and "COHORT" variables (A) regardless of age group or (B) also according to age group.

Global TE expression was measured in each karyotype (x-axis) as the proportion of TE read counts among all read counts (TEs and genes) (y-axis). In this calculation, read counts were used after DESeq2 normalization ("normalized counts") to remove any depth sequencing bias. Each dot represents one individual. Dots are colored according to sex. Sex variable has two levels: female, male. COHORT variable has two levels: Organ Donor (OPO), Postmortem.

(**A**) Proportion of TE read counts among all read counts was represented by sex for Organ Donor (OPO) individuals (top graph), for Postmortem individuals (middle graph), and without distinguishing them (bottom graph: "all").
Concerning the top and middle graphs, proportion of TE read counts among all read counts was modelized using a linear model with age group (5 levels: [20-30],]30-40],]40-50],]50-60], and]60-70]), sex, COHORT and interaction between the last two as independent variables. From this model, not significant p-values were obtained testing the age group adjusted effect of sex on proportion of TE read counts among all read counts in Organ Donor (OPO) individuals and in Postmortem individuals.
Concerning the bottom graph, proportion of TE read counts among all read counts was modelized using a linear model with age group, sex and COHORT as independent variables. From this model, the p-value testing the effect of sex on proportion of TE read counts among all read counts adjusted on age group and COHORT was not significant.

(**B**) A plot was drawn and an ANOVA model comparing proportion of TE read counts among all read counts across age groups was used in each sex*COHORT level (female*Organ Donor (OPO), male*Organ Donor (OPO), female*Postmortem, and male*Postmortem). P-values testing the effect of the "age group" variable were not significant in each of the cases considered. P-value of age group effect adjusted on COHORT and sex was also not significant (p-value = 0.198) using an ANOVA model in the whole dataset.

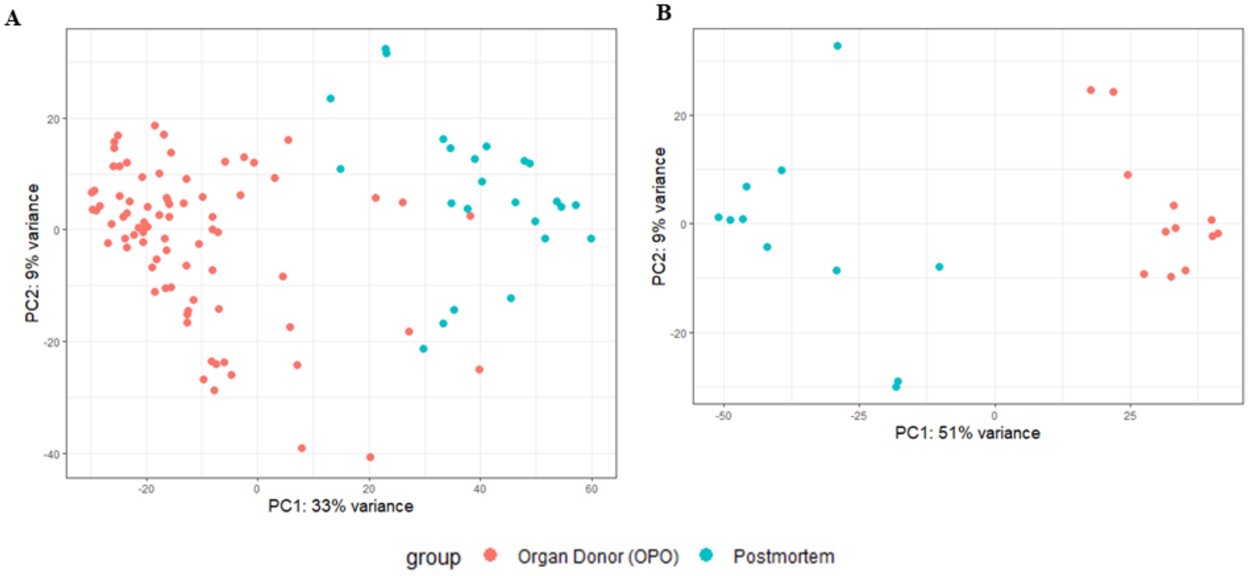

Fig V. PCA analysis of GTEx expression data performed on the 500 genes explaining most of the variance for the identification of confounding factors considering (A) all of the 104 individuals with optimal data for use in the analysis after application of selection criteria and (B) only individuals in the 60-70-year-old group (n = 23/104).

Each dot represents one individual. Dots are colored according to the two levels of the “COHORT” variable: “Organ Donor (OPO)” and “Postmortem”. PC: Principal Component.

Supplementary tables

Table A. List of sequenced individuals and details on RNA-seq data in the gonosome aneuploidy dataset.

Identifier	Karyotype	Sex	Age (years)	Batch	Total number of read pairs	Pseudo-alignment rate Kallisto (genes)	Alignment rate TEcount (TE)
Txx20a	46,XX	Female	63	1	184223353	59.94%	19.35%
Txx21b	46,XX	Female	35	2	132285589	60.39%	15.65%
Txx22a	46,XX	Female	32	1	205687025	62.32%	17.74%
Txx23a	46,XX	Female	29	1	169410086	63.82%	17.52%
Txx24a	46,XX	Female	25	1	170479762	57.81%	16.40%
Txx25b	46,XX	Female	51	2	167418680	48.30%	16.08%
Txxy20b	47,XXY*	Male	30	2	153002185	53.52%	12.36%
Txxy22a	47,XXY	Male	20	1	185535783	42.63%	14.59%
Txxy23a	47,XXY	Male	34	1	183001944	47.65%	14.72%
Txxy30a	47,XXY	Male	18	2	177627640	49.05%	11.20%
Txxy31a	47,XXY	Male	17	2	149962790	45.56%	11.36%
Txxy32a	47,XXY	Male	30	2	156062964	52.10%	13.37%
Txxy33a	47,XXY	Male	20	2	171080372	50.14%	11.57%
Txxyb	47,XXY	Male	18	1	173366155	58.29%	14.32%
Txy20a	46,XY	Male	27	1	186765891	64.23%	17.97%
Txy21a	46,XY	Male	56	1	185880218	56.05%	14.16%
Txy22a	46,XY	Male	27	1	188136209	62.55%	17.84%
Txy23b	46,XY	Male	26	2	143693391	44.53%	11.26%
Txy24a	46,XY	Male	50	1	174258860	53.70%	15.90%
Txy25b	46,XY	Male	63	2	134285944	53.05%	17.38%
Txyy20a	47,XYY	Male	39	1	184316111	46.90%	14.69%

Txyy30a	47,XYY	Male	34	2	174829021	40.65%	12.61%
Txyy31a	47,XYY*	Male	30	2	161160366	46.24%	10.91%
Txyyb	47,XYY	Male	15	1	188386148	54.03%	14.27%

TE: transposable elements.
*All 47,XXY males and 47,XYY males had homogenous 47,XXY or 47,XYY karyotype respectively except for Txxy20b individual (95% of mitoses were XXY, 3% XY, and 2% XXXY) and Txyy31a individual (97% of mitoses were XXY and 3% XY).

Table B. Percentages of significantly differentially expressed protein-coding genes after batch adjustment in the gonosome aneuploidy dataset in each of the 6 pairwise comparisons of karyotypes and according to the gene localization: total (n = 19947 genes), autosomes (n = 19035 genes), X chromosome (n = 851 genes), or Y chromosome (n = 66 genes including PAR, n = 48 genes excluding PAR)

Variation	In XY compared to XX	In XXY compared to XX	In XYY compared to XX	In XXY compared to XY	In XYY compared to XY	In XYY compared to XXY
Upregulated (%) – Total (19947 genes)	19 (0.10%)	726 (3.64%)	518 (2.60%)	104 (0.52%)	30 (0.15%)	35 (0.18%)
Downregulated (%) – Total (19947 genes)	18 (0.09%)	1167 (5.85%)	992 (4.97%)	37 (0.19%)	28 (0.14%)	15 (0.08%)
Upregulated (%) – Autosomes (19035 genes)	5 (0.03%)	692 (3.64%)	480 (2.52%)	87 (0.46%)	19 (0.10%)	29 (0.15%)
Downregulated (%) – Autosomes (19035 genes)	4 (0.02%)	1113 (5.85%)	936 (4.92%)	36 (0.19%)	26 (0.14%)	11 (0.06%)
Upregulated (%) – Xchr (851 genes)	1 (0.12%)	21 (2.47%)	25 (2.94%)	17 (2.00%)	6 (0.71%)	0 (0%)
Downregulated (%) – Xchr (851 genes)	14 (1.65%)	41 (4.82%)	43 (5.05%)	1 (0.12%)	1 (0.12%)	4 (0.47%)
Upregulated (%) – Ychr including PAR (66 genes)	13 (19.70%)	19 (28.79%)	20 (30.30%)	3 (4.55%)	9 (13.64%)	6 (9.09%)
Downregulated (%) – Ychr including PAR (66 genes)	0 (0%)	0 (0%)	0 (0%)	0 (0%)	0 (0%)	0 (0%)
Upregulated (%) – Ychr excluding PAR (48 genes)	13 (27.08%)	13 (27.08%)	13 (27.08%)	0 (0%)	5 (10.42%)	6 (12.50%)
Downregulated(%) – Ychr excluding PAR (48 genes)	0 (0%)	0 (0%)	0 (0%)	0 (0%)	0 (0%)	0 (0%)

PAR: pseudo-autosomal region.

Table C. All the significantly differentially expressed protein-coding genes according to karyotype localized on the Y chromosome in the gonosome aneuploidy dataset likelihood-ratio test.

ENSG identifier	Gene name	Gene description	In PAR ?
ENSG00000114374	*USP9Y*	ubiquitin specific peptidase 9 Y-linked	No
ENSG00000067048	*DDX3Y*	DEAD-box helicase 3 Y-linked	No
ENSG00000183878	*UTY*	ubiquitously transcribed tetratricopeptide repeat containing, Y-linked	No
ENSG00000012817	*KDM5D*	lysine demethylase 5D	No
ENSG00000099725	*PRKY*	protein kinase Y-linked (pseudogene)	No
ENSG00000129824	*RPS4Y1*	ribosomal protein S4 Y-linked 1	No
ENSG00000198692	*EIF1AY*	eukaryotic translation initiation factor 1A Y-linked	No
ENSG00000067646	*ZFY*	zinc finger protein Y-linked	No
ENSG00000154620	*TMSB4Y*	thymosin beta 4 Y-linked	No
ENSG00000165246	*NLGN4Y*	neuroligin 4 Y-linked	No
ENSG00000280969	*RPS4Y2*	ribosomal protein S4 Y-linked 2	No
ENSG00000169084	*DHRSX*	dehydrogenase/reductase X-linked	Yes
ENSG00000169093	*ASMTL*	acetylserotonin O-methyltransferase like	Yes
ENSG00000197976	*AKAP17A*	A-kinase anchoring protein 17A	Yes
ENSG00000169789	*PRY*	PTPN13 like Y-linked	No
ENSG00000169807	*PRY2*	PTPN13 like Y-linked 2	No
ENSG00000182378	*PLCXD1*	phosphatidylinositol specific phospholipase C X domain containing 1	Yes
ENSG00000214717	*ZBED1*	zinc finger BED-type containing 1	Yes
ENSG00000182162	*P2RY8*	P2Y receptor family member 8	Yes
ENSG00000124334	*IL9R*	interleukin 9 receptor	Yes

Top genes are those with the lowest adjusted p-values.
PAR: pseudo-autosomal region.

Table D. All the significantly differentially expressed protein-coding genes according to karyotype localized on the X chromosome in the gonosome aneuploidy dataset likelihood-ratio test.

ENSG identifier	Gene name	Gene description
ENSG00000184368	MAP7D2	MAP7 domain containing 2
ENSG00000089472	HEPH	hephaestin
ENSG00000169084	DHRSX	dehydrogenase/reductase X-linked
ENSG00000169093	ASMTL	acetylserotonin O-methyltransferase like
ENSG00000169249	ZRSR2	zinc finger CCCH-type, RNA binding motif and serine/arginine rich 2
ENSG00000011201	ANOS1	anosmin 1
ENSG00000188937	NYX	nyctalopin
ENSG00000198034	RPS4X	ribosomal protein S4 X-linked
ENSG00000130021	PUDP	pseudouridine 5'-phosphatase
ENSG00000005889	ZFX	zinc finger protein X-linked
ENSG00000173674	EIF1AX	eukaryotic translation initiation factor 1A X-linked
ENSG00000197976	AKAP17A	A-kinase anchoring protein 17A
ENSG00000147050	KDM6A	lysine demethylase 6A
ENSG00000178947	SMIM10L2A	small integral membrane protein 10 like 2A
ENSG00000182378	PLCXD1	phosphatidylinositol specific phospholipase C X domain containing 1
ENSG00000069509	FUNDC1	FUN14 domain containing 1
ENSG00000126012	KDM5C	lysine demethylase 5C
ENSG00000006757	PNPLA4	patatin like phospholipase domain containing 4
ENSG00000183943	PRKX	protein kinase cAMP-dependent X-linked catalytic subunit
ENSG00000165704	HPRT1	hypoxanthine phosphoribosyltransferase 1
ENSG00000130962	PRRG1	proline rich and Gla domain 1
ENSG00000180879	SSR4	signal sequence receptor subunit 4

ENSG00000186462	NAP1L2	nucleosome assembly protein 1 like 2
ENSG00000184515	BEX5	brain expressed X-linked 5
ENSG00000102287	GABRE	gamma-aminobutyric acid type A receptor subunit epsilon
ENSG00000214717	ZBED1	zinc finger BED-type containing 1
ENSG00000147123	NDUFB11	NADH:ubiquinone oxidoreductase subunit B11
ENSG00000182162	P2RY8	P2Y receptor family member 8
ENSG00000086712	TXLNG	taxilin gamma
ENSG00000126756	UXT	ubiquitously expressed prefoldin like chaperone
ENSG00000155966	AFF2	ALF transcription elongation factor 2
ENSG00000196459	TRAPPC2	trafficking protein particle complex subunit 2
ENSG00000257529	RPL36A-HNRNPH2	RPL36A-HNRNPH2 readthrough
ENSG00000198088	NUP62CL	nucleoporin 62 C-terminal like
ENSG00000182712	CMC4	C-X9-C motif containing 4
ENSG00000089289	IGBP1	immunoglobulin binding protein 1
ENSG00000047230	CTPS2	CTP synthase 2
ENSG00000068985	PAGE1	PAGE family member 1
ENSG00000180964	TCEAL8	transcription elongation factor A like 8
ENSG00000277203	None	novel transcript
ENSG00000125354	SEPTIN6	septin 6
ENSG00000065923	SLC9A7	solute carrier family 9 member A7
ENSG00000186310	NAP1L3	nucleosome assembly protein 1 like 3
ENSG00000205542	TMSB4X	thymosin beta 4 X-linked
ENSG00000183837	PNMA3	PNMA family member 3
ENSG00000188706	ZDHHC9	zinc finger DHHC-type palmitoyltransferase 9
ENSG00000172943	PHF8	PHD finger protein 8

ENSG00000147403	RPL10	ribosomal protein L10
ENSG00000198932	GPRASP1	G protein-coupled receptor associated sorting protein 1
ENSG00000124334	IL9R	interleukin 9 receptor
ENSG00000186871	ERCC6L	ERCC excision repair 6 like, spindle assembly checkpoint helicase
ENSG00000123595	RAB9A	RAB9A, member RAS oncogene family
ENSG00000183479	TREX2	three prime repair exonuclease 2
ENSG00000094631	HDAC6	histone deacetylase 6
ENSG00000165775	FUNDC2	FUN14 domain containing 2
ENSG00000176896	TCEANC	transcription elongation factor A N-terminal and central domain containing
ENSG00000158423	RIBC1	RIB43A domain with coiled-coils 1
ENSG00000169239	CA5B	carbonic anhydrase 5B
ENSG00000125356	NDUFA1	NADH:ubiquinone oxidoreductase subunit A1
ENSG00000079482	OPHN1	oligophrenin 1
ENSG00000215301	DDX3X	DEAD-box helicase 3 X-linked
ENSG00000169057	MECP2	methyl-CpG binding protein 2
ENSG00000232119	MCTS1	MCTS1 re-initiation and release factor
ENSG00000029993	HMGB3	high mobility group box 3
ENSG00000102053	ZC3H12B	zinc finger CCCH-type containing 12B
ENSG00000198918	RPL39	ribosomal protein L39
ENSG00000284987	None	novel transcript
ENSG00000160131	VMA21	vacuolar ATPase assembly factor VMA21
ENSG00000241343	RPL36A	ribosomal protein L36a
ENSG00000131174	COX7B	cytochrome c oxidase subunit 7B
ENSG00000238269	PAGE2B	PAGE family member 2B
ENSG00000102225	CDK16	cyclin dependent kinase 16

ENSG00000156709	AIFM1	apoptosis inducing factor mitochondria associated 1
ENSG00000130741	EIF2S3	eukaryotic translation initiation factor 2 subunit gamma
ENSG00000185515	BRCC3	BRCA1/BRCA2-containing complex subunit 3
ENSG00000173275	ZNF449	zinc finger protein 449
ENSG00000130826	DKC1	dyskerin pseudouridine synthase 1

Top genes are those with the lowest adjusted p-values.

Table E. Differentially expressed TE subfamilies in samples with different karyotypes among the 1246 explored in the gonosome aneuploidy dataset.

Variation	In XY compared to XX	In XXY compared to XX	In XYY compared to XX	In XXY compared to XY	In XYY compared to XY	In XYY compared to XXY
Upregulated TE subfamilies	7 subfamilies (0.6%):<br><br>LTR22B2<br>MSTB1-int<br>LTR19-int*<br>MER70B<br>LTR19A<br>MER51C<br>LTR25-int	79 subfamilies (6.3%):<br><br>LTR22B2<br>LTR19-int*<br>MSTB1-int<br>LTR19A<br>HERV9-int<br>MER51C<br>LTR25-int<br>MER61F<br>HERV3-int<br>LTR15*<br>LTR5B<br>LTR68<br>MER115<br>LTR1F1*<br>THE1A-int<br>MER70B<br>MER126<br>LTR13<br>**HERVL18-int**<br>LTR10G<br>**UCON46**<br>**hAT-N1_Mam**<br>Charlie15a<br>MER91B<br>LTR5_Hs<br>ORSL<br>MER52D*<br>LTR10F<br>LTR19B<br>PRIMA41-int<br>LTR1F2<br>MER96B<br>HERV15-int<br>LTR35<br>LTR14<br>MLT1G<br>MLT1M<br>MER50C<br>MER21B<br>LTR53<br>MER21A<br>ERVL-int<br>MER34C2<br>MLT1A-int<br>**LTR18A**<br>**MamRep38**<br>LTR22B<br>MER9a1<br>MER68-int<br>MER30B*<br>LTR12B<br>LTR4<br>ERV3-16A3_LTR	43 subfamilies (3.5%):<br><br>LTR22B2<br>MSTB1-int<br>LTR19-int*<br>LTR19A<br>MER51C<br>MER61F<br>HERV9-int<br>LTR25-int<br>MER70B<br>LTR12B<br>LTR68<br>HERV3-int<br>LTR40A1<br>LTR15*<br>HERVK22-int<br>LTR10F<br>LTR25<br>MER9a1<br>MER52D*<br>LTR1F1*<br>LTR5B<br>MER50C<br>LTR13<br>MER41D*<br>HERV1_LTRe<br>LTR4<br>MamGypLTR3a<br>LTR5_Hs<br>LTR19B<br>MLT1N2<br>THE1A-int<br>MER92B<br>LTR66<br>**X7C_LINE**<br>MER4A1<br>MER34C2<br>Charlie22a<br>MER91B<br>LTR17<br>Ricksha_b<br>MER21B<br>HERVL66-int<br>MLT2B2	0 subfamily	0 subfamily	0 subfamily

		MamTip3<br>SVA_D<br>**MLT1C2-int**<br>MER52A<br>**UCON86***<br>LTR22C0*<br>LTR26D<br>MER74B*<br>**X7C_LINE**<br>LTR66<br>HERVK22-int<br>MER66B<br>MSTD-int<br>HERV1_LTRe<br>THE1B-int<br>HERVK3-int<br>MLT1N2<br>**MER51E***<br>MER117<br>**UCON89**<br>LTR23-int<br>MLT1E3<br>LTR40A1<br>LTR3B_<br>MER91A<br>MER92B				
Downregulated TE subfamilies	1 subfamily (0.1%):<br><br>**UCON75**	16 subfamilies (1.3%):<br><br>HERVH-int<br>LTR46-int<br>**HERVH48-int**<br>HERVFH21-int*<br>UCON132b<br>**MER41G**<br>Alu<br>**LTR1C**<br>LTR10B1<br>LTR37B<br>**UCON5**<br>FordPrefect*<br>LTR7Y*<br>**UCON81**<br>LTR73<br>**UCON1**	17 subfamilies (1.4%):<br><br>HERVH-int<br>**HERVH48-int**<br>UCON132b<br>HERV1_LTRc<br>LTR46-int<br>**MER41G**<br>HERVFH21-int*<br>AluYj4<br>FordPrefect*<br>AluYb9<br>**UCON5**<br>LTR10B1<br>LTR37B<br>**MER96**<br>**UCON1**<br>HERV1_I-int<br>Alu	0 subfamily	0 subfamily	0 subfamily

TE subfamily names are classified according to adjusted p-values in ascending order. The colored names are names found in at least three comparisons.
Underlined TE subfamily names are those enriched in the Y chromosome (binomial test adjusted p-value < 0.05 and number of observed copies located on Y chromosome > expected). Bold TE subfamily names are TE family with no copy located in the Y chromosome in the TE sequence reference file.

* found significantly differentially expressed in any comparison in the filtered GTEx dataset.

Variation is upregulated if TE subfamily is upregulated in the first karyotype (e.g. XYY in the “XYY karyotype vs XX karyotype” comparison) compared to the second (e.g. XX in the “XYY karyotype vs XX karyotype”

comparison). Variation is downregulated if TE subfamily is downregulated in the first karyotype (e.g. XYY in the "XYY karyotype vs XX karyotype" comparison) compared to the second (e.g. XX in the "XYY karyotype vs XX karyotype" comparison).

See **S5 Data** for number of TE subfamilies upregulated and downregulated per TE family or TE superfamily/order/class in all the pairwise karyotype comparisons.

Table F. Overlap between intragenic regions of differentially expressed genes and differentially expressed TE subfamilies

Pairwise karyotype comparison	Overlap between	Expected proportion*	Observed proportion**	P-value (exact Binomial test)
XY are compared to XX	Upregulated TEs and upregulated genes	0.00340 (206/60564)	0.04762 (2/42)	0.009
XY are compared to XX	Downregulated TEs and downregulated genes	0.00007 (4/60564)	0 (0/35)	1
XXY are compared to XX	Upregulated TEs and upregulated genes	0.13252 (8026/60564)	0.20994 (228/1086)	<0.001
XXY are compared to XX	Downregulated TEs and downregulated genes	0.03537 (2142/60564)	0.05473 (88/1608)	<0.001
XYY are compared to XX	Upregulated TEs and upregulated genes	0.06681 (4046/60564)	0.09986 (71/711)	<0.001
XYY are compared to XX	Downregulated TEs and downregulated genes	0.05417 (3281/60564)	0.09098 (123/1352)	<0.001

TE: transposable element.

*Expected proportion corresponds to genes containing at least one TE copy from one of the upregulated TE subfamilies among all the 60564 genes or to genes containing at least one TE copy from one of the downregulated TE subfamilies among all the 60564 genes according to what is indicated in the “overlap between” column (details of the calculation are in brackets).

**Observed proportion corresponds to upregulated genes containing at least one TE copy from one of the upregulated TE subfamilies among all the upregulated genes or to downregulated genes containing at least one TE copy from one of the downregulated TE subfamilies among all the downregulated genes according to what is indicated in the “overlap between” column (details of the calculation are in brackets).

Table G. Repartition of GTEx individuals according to age group and sex in the filtered GTEx dataset (no disease group).

Age group (years)	[20-30]	]30-40]	]40-50]	]50-60]	]60-70]	Total
Females	7	3	11	6	8	35
Males	7	8	13	13	4	45
Total	14	11	24	19	12	80

Table H. Repartition of GTEx individuals according to age group and sex in the filtered GTEx dataset (group including individuals with current or antecedents of cancer or cardiovascular diseases).

Age group (years)	[20-30]	]30-40]	]40-50]	]50-60]	]60-70]	Total
Females	7	4	12	8	14	45
Males	7	9	15	20	6	57
Total	14	13	27	28	20	102

Table I. Differentially expressed TE subfamilies among the 1246 explored in the filtered GTEx (no disease group) dataset between all the pairwise comparisons of age groups in each sex.

Age group (years)	Variation	In males	In females	Overall adjusted on sex
In]30-40] compared to [20-30]	Upregulated	MER51E*<br>MER30B*<br>LTR1F1*<br>MER74C<br>HERV35I-int<br>MER110A<br>LTR24<br>FordPrefect*<br>MLT1H1-int<br>LTR1A1<br>Looper<br>LTR9C<br>MER65C<br>LTR9B	0	0
	Downregulated	0	0	0
In]40-50] compared to [20-30]	Upregulated	0	0	0
	Downregulated	0	0	0
In]50-60] compared to [20-30]	Upregulated	0	0	HERV35I-int
	Downregulated	LTR48B	0	LTR48B
In]60-70] compared to [20-30]	Upregulated	0	0	LTR10E<br>MLT2B5<br>Tigger11a<br>MER121<br>SVA_A
	Downregulated	0	0	0
In]40-50] compared to]30-40]	Upregulated	0	0	LTR35B
	Downregulated	HSMAR2<br>MER110A<br>L1M2c<br>MER51E*<br>MLT1H1-int<br>L1PA12<br>LTR24<br>HUERS-P1-int<br>LTR1F1*	0	HSMAR2<br>L1M2c<br>MER41D*
In]50-60] compared to]30-40]	Upregulated	0	0	0
	Downregulated	MER51E*<br>L1M2c<br>UCON49<br>MER110A<br>UCON86*<br>LTR24<br>MER74C<br>MER74B*	0	L1M2c<br>MER51E*<br>LTR24<br>MER74C<br>UCON38<br>MER74B*<br>UCON49<br>Arthur1C

		Arthur1C<br>UCON38<br>L1P4e<br>MER57C1<br>HERVFH21-int*<br>HSMAR2<br>LTR9C<br>MER8<br>L4_A_Mam		MER8<br>LTR86C<br>L4_A_Mam<br>MER57C1<br>LTR9C<br>MER110A<br>LTR22C0*
In]60-70] compared to]30-40]	Upregulated	0	0	MER99
	Downregulated	MLT1E1A-int<br>MER74C<br>LTR15*<br>MER110A	0	MER74C<br>MER51E*<br>HERVFH21-int*<br>LTR21A<br>MER65C<br>LTR24<br>LTR19-int*<br>LTR81
In]50-60] compared to]40-50]	Upregulated	0	0	0
	Downregulated	0	0	0
In]60-70] compared to]40-50]	Upregulated	0	0	0
	Downregulated	0	0	0
In]60-70] compared to]50-60]	Upregulated	0	0	MER51-int<br>L1M3e<br>ORSL-2a<br>MamGyp-int
	Downregulated	0	0	0

TE subfamily names are classified according to adjusted p-values in ascending order. The coloured names are names found in at least three comparisons. Note that none of these TE subfamilies were significantly differentially expressed according to the sex variable. Variation is upregulated if TE subfamily is upregulated in the first age group (e.g.]30-40] in the "]30-40] vs [20-30]" comparison) compared to the second (e.g. [20-30] in the "]30-40] vs [20-30]" comparison). Variation is downregulated if TE subfamily is downregulated in the first age group (e.g.]30-40] in the "]30-40] vs [20-30]" comparison) compared to the second (e.g. [20-30] in the "]30-40] vs [20,30]" comparison). Underlined TE subfamilies correspond to those for which we found a significant interaction indicating a log2-fold change in males compared to females that was higher in the]30-40] age group than in the [20-30] age group: MER74C, MER110A.

* found significantly differentially expressed in some pairwise comparisons of karyotypes in the gonosome aneuploidy dataset.

Table J. Differentially expressed TE subfamilies among the 1246 explored in the filtered GTEx dataset (no disease group) between males and females in each age group.

Age group (years)	Upregulated TE subfamilies in males compared to females	Downregulated TE subfamilies in males compared to females
[20-30]	0	0
]30-40]	0	0
]40-50]	MER52D*<br>LTR27D	0
]50-60]	MER52D*<br>LTR27D	0
]60-70]	0	0
Overall adjusted on age group	MER52D*<br>LTR27D<br>L1MDb	0

TE subfamily names are classified according to adjusted p-values in ascending order. The colored names are names found in at least two comparisons.
Note that none of these TE subfamilies were significantly differentially expressed according to the age group variable. All of these 3 subfamilies had at least one copy on the Y chromosome and one copy on the X chromosome, but none are enriched on the Y chromosome.

* found significantly differentially expressed in some pairwise comparisons of karyotypes in the gonosome aneuploidy dataset.

Table K. Differentially expressed TE subfamilies among the 1246 explored in the filtered GTEx (group including subjects with current or antecedents of cancer or cardiovascular diseases) dataset between all the pairwise comparisons of age groups in each sex.

Age group (years)	Variation	In males	In females	Overall adjusted on sex
In]30-40] compared to [20-30]	Upregulated	MER51E*<br>MER30B*<br>MER74C<br>MER110A<br>Looper<br>LTR1F1*<br>HERV35I-int<br>LTR9C<br>LTR24<br>MLT1H1-int	0	0
	Downregulated	0	0	0
In]40-50] compared to [20-30]	Upregulated	0	0	0
	Downregulated	0	0	0
In]50-60] compared to [20-30]	Upregulated	0	0	HERV35I-int<br>MamRep4096<br>LTR1C3<br>L1MD1
	Downregulated	0	0	MER65-int
In]60-70] compared to [20-30]	Upregulated	0	Tigger11a	LTR10E<br>Tigger11a<br>MLT2B5<br>SVA_A<br>HERV9N-int<br>MER121<br>HERVI-int<br>MER30
	Downregulated	0	0	MER65-int
In]40-50] compared to]30-40]	Upregulated	0	0	0
	Downregulated	HSMAR2<br>MER110A<br>MER51E*<br>L1M2c<br>MLT1H1-int<br>LTR24<br>L1PA12<br>Eulor6E<br>MER74C<br>LTR15*<br>MER30B*<br>L1MA7<br>L1MD<br>LTR86A2<br>L1PB2<br>Arthur1C<br>L4_A_Mam	0	HSMAR2

		LTR37-int		
In]50-60] compared to]30-40]	Upregulated	0	0	0
	Downregulated	MER51E*<br>MER74C<br>L1M2c<br>LTR24<br>MER74B*<br>HSMAR2<br>MER113B<br>HERVFH21-int*,†<br>MER8<br>UCON86*<br>L4_A_Mam<br>MER110A<br>MER41D*<br>MER53<br>MLT1H1-int<br>UCON38<br>L1ME5<br>Arthur1C<br>L1M3e<br>L1ME3C<br>L1PA12<br>Looper<br>L1P2<br>LTR9C<br>L1MA7<br>MARNA<br>MER30B*<br>UCON49<br>L5<br>Cheshire<br>L1PB2<br>LTR38	0	L1M2c<br>UCON38<br>LTR24<br>Helitron1Nb_Mam<br>MER74B*<br>L1M3e<br>MER8<br>HSMAR2<br>L5<br>L4_A_Mam<br>MER41D*
In]60-70] compared to]30-40]	Upregulated	0	0	0
	Downregulated	MER74C<br>MLT1E1A-int<br>MER110A<br>MER51E<br>LTR5A<br>LTR24<br>LTR38<br>HERVFH21-int*,†<br>L1M2c<br>LTR21A<br>L1PA12<br>ERV24_Prim-int<br>LTR15*	0	LTR21A<br>L1M2c<br>HERVFH21-int*,†<br>LTR47A2
In]50-60] compared to]40-50]	Upregulated	0	0	0
	Downregulated	0	0	0
In]60-70] compared to]40-50]	Upregulated	0	0	0
	Downregulated	0	0	0

In]60-70] compared to]50-60]	Upregulated	0	0	0
	Downregulated	0	0	0

TE subfamily names are classified according to adjusted p-values in ascending order. The colored names are names found in at least three comparisons. Variation is upregulated if TE subfamily is upregulated in the first age group (e.g.]30-40] in the "]30-40] vs [20-30]" comparison) compared to the second (e.g. [20-30] in the "]30-40] vs [20-30]" comparison). Variation is downregulated if TE subfamily is downregulated in the first age group (e.g.]30-40] in the "]30-40] vs [20-30]" comparison) compared to the second (e.g. [20-30] in the "]30-40] vs [20-30]" comparison).

Underlined TE subfamilies correspond to those for which we found a significant interaction: MER74C, MER110A, and MER51E showed a log2-fold change in males compared to females that was higher in the]30-40] age group than in the [20-30] age group; MER74C and MER110A showed a log2-fold change in males compared to females that was lower in the]60-70] age group than in the]30-40] age group; MER110A showed a log2-fold change in males compared to females that was lower in the]40-50] age group than in the]30-40] age group.

* found significantly differentially expressed in some pairwise comparisons of karyotypes in the gonosome aneuploidy dataset.
† found significantly differentially expressed in males vs females in some age groups of the filtered GTEx dataset in the group including subjects with current or antecedents of cancer of cardiovascular diseases.

Table L. Differentially expressed TE subfamilies among the 1246 explored in the filtered GTEx dataset between males on females in each age group, in the group including subjects with current or antecedents of cancer of cardiovascular diseases.

Age group (years)	Upregulated TE subfamilies in males compared to females	Downregulated TE subfamilies in males compared to females
[20-30]	0	0
]30-40]	MER52D*	0
]40-50]	MER52D*	0
]50-60]	MER52D*<br>LTR27D	0
]60-70]	MER52D*	0
Overall adjusted on age group	MER52D*<br>LTR27D<br>L1MDb<br>Tigger3a	HERVFH21-int*,†<br>LTR21B<br>LTR19-int*

TE subfamily names are classified according to adjusted p-values in ascending order. The colored names are names found in at least two comparisons.

* found significantly differentially expressed in some pairwise comparisons of karyotypes in the gonosome aneuploidy dataset.

† found significantly differentially expressed in some pairwise comparisons of age groups in the filtered GTEx dataset in the group including subjects with current or antecedents of cancer of cardiovascular diseases.

Table M. Repartition of GTEx individuals according to age group and sex in the large GTEx dataset.

Age group (years)	[20-30]	]30-40]	]40-50]	]50-60]	]60-70]	Total
Females	15	5	30	25	30	105
Males	15	26	39	68	61	209
Total	30	31	69	93	91	314
