# Supplementary material for: Transposable element expression is associated with sex chromosome number in humans
Source: PLoS Genet. 2025 Jun 26;21(6):e1011668. doi: 10.1371/journal.pgen.1011668 (PMC12225981; doi:10.1371/journal.pgen.1011668)
Supplement: Attachment — Submitted filename: PloS Gen Review 9.24.pdf [file pgen.1011668.s009.pdf]

Review for "Transposable element expression with variation in sex chromosome number: insights into a toxic Y effect on human longevity"

PLoS Genetics September 2024

Teoli et al. seek to answer whether Y chromosome copy number is associated with increased transposable element (TE) expression in humans. The authors perform gene expression profiling (RNA-seq) on whole blood samples from study participants with four different karyotypes: XX, XY, XXY, and XYY, allowing them to measure the effect of Y chromosome dosage on gene and TE expression. Gene expression across the four groups is examined first. Despite clear separation on the PCA plot, XY vs XX groups show only a small number of differentially expressed (DE) genes. However, XXY vs XX and XYY vs XX show large numbers of differentially expressed genes, while XY vs XXY or XYY show a moderate number of differentially expressed genes. The authors then quantify all TEs and TE family/sub-family expression from the same RNA-seq dataset, using TETools. For the "all TEs" group, and all TE families except DNA transposons, expression levels seem to show an overall trend of increasing as Y chromosome copy number increases. However, the differences between karyotypes only reach statistical significance in certain comparisons and TE families. No TE family has significantly higher expression in the XY group compared to XX. For LTR and SVA TEs, XYY has significantly higher expression than the XY group. Several TE subfamilies are identified which do show statistically significant increases in XY vs XX, and XYY vs XY, which are primarily LTR and HERV subfamilies. As TE expression is thought to increase with age, the authors also measure TE expression in a separate, larger cohort of XX and XY subjects of ages 20-70 years old. Contrary to the authors initial hypothesis, old XY subjects do not have higher TE expression than old XX subjects, nor was there a significant change in overall TE ("all TEs") expression with age in either sex. In this second cohort, three TE sub-families showed higher expression in XY vs XX subjects across multiple age groups, while no sub-family showed higher expression in XX vs XY. The primary conclusion of the study is that the number of Y chromosomes is associated with increased TE expression, particularly within certain TE sub-families.

Overall, the study provides important and novel experimental insight into the effect of Y chromosome copy number on TE expression. To my knowledge, no prior similar studies have been conducted in human subjects with sex chromosome aneuploidies (though similar studies have been performed in *Drosophila*, which the authors discuss). The study is somewhat limited in scope, as all of the figures are generated from RNA-seq data from two datasets, one of which has a small sample size. Still, the authors analyze these datasets thoroughly, as seen in the extensive supplementary materials. The main finding that Y chromosome copy number is associated with higher TE expression in humans is convincing and should be of interest and value to the field. However, there are a number of concerns that should be addressed before I can recommend for publication. Thus, my overall recommendation is for the authors to address these concerns and re-submit.

Major concerns:

1. In general the writing is clear, but there are so many comparisons (often feeling somewhat random, e.g. focusing on XX vs XY one paragraph and XX vs XYY the next) and supplementary analyses, the main points can be easily lost or missed. The manuscript would benefit from editing to streamline and improve clarity. Specific things that would help include:
    1. establish and state the primary comparisons of interest for testing the main hypotheses of the study (I believe to be XY vs XX and XYY vs XY, but the authors should state clearly),
    2. consistent presentation of comparisons (i.e. consistently discuss the same comparisons in the same order, e.g. XX vs XY, then XY vs XYY)
    3. the addition of conclusions to end of paragraphs in results section.

2. It seems a little strange that XX and XY groups separate well on PCA (Fig S2) but have very few differentially expressed genes that reach statistical significance between the two groups. Is this consistent with other blood RNA-seq studies?

3. The manuscript repeatedly says the Y chromosome is enriched for TEs, but don't provide any sort of quantification for this. Please provide some type of numerical frame of reference for TE enrichment on Y chromosome. For example, see Erlandsson, Wilson, Paabo 2000 reference from the manuscripts reference list. Based on this information, what would be the predicted impact of Y chromosome copy number on % increase in TE expression?

4. Lines 216 and 421: the authors note twice that addition of an extra X chromosome (ie XXY) is also associated with higher TE levels, "probably/likely" due to "increased genomic material" and, consequently, TE load. What do the authors mean exactly by "increased genomic material?" Maybe I am misunderstanding this point, but I believe RNA-seq library prep generally uses a standardized amount of input RNA from all samples (it is not clear from the methods if the authors did this), which would correct for differences in genomic material. In addition, RNA-seq analysis is based on relative expression and thus, unless a spike-in is used, differences in expression based on initial input are lost during normalization. Could the authors clarify this point and the methods used for library prep?

5. Are there any other reasons besides "increased genomic material" that might explain why XXY are often found to have (a trend towards) higher TE expression than XY?

6. In relation to Figure 2 and S12, could the authors make a table for each main TE family, showing the number and percent of TE sub-families which were:
    1. significantly higher in XY vs XX
    2. significantly higher in XYY vs XY
    3. significantly lower in in XY vs XX
    4. significantly lower in XYY vs XY

This would provide helpful context to the results shown in Figure 2 and would be easier to digest than Figure S12.

7. Only 1 (MER52D) of 3 TE sub-families found to be significantly higher in XY than XX from the second cohort in Figure 4 also show up as significantly higher in XY vs XX in Figure 2 and Figure S12. Can the authors comments on this – does it suggest a lack of agreement of consistency between datasets?

8. Figure S14 shows roughly the same pattern for TE expression across the 4 karyotypes regardless of whether the TE sub-families are enriched, depleted, not enriched or depleted, or not present at all on Y or X chromosomes. Could the authors discuss whether this weakens the central conclusion that increased TE expression with increasing Y copy number is due to a high number of TEs on the Y chromosome?

9. Line 276 "…the proportion of copies of this TE subfamily carried by the Y chromosome increased, was also more pronounced in the Y-enriched TE subfamily group than in the not Y-enriched TE subfamily group (Fig. S13B)." This sentence is confusing and could be written more clearly. Given the accompanying Figure S13B, I am not sure what the point or conclusion is.

10. Line 306 "upstream regions" should be defined, i.e. in base pairs from TSS.

Minor points:

- The discussion would benefit from adding a reference to De Cecco et al., 2019 (Nature volume 566, pages73–78) when discussing mechanisms by which increased TE expression can be harmful without transposition. Gorbunova et al., 2021 (Nature volume 596, pages43–53) may also be worth adding.

- Line 166 of supplementary material for Fig S14 "(A) Y-enriched TE subfamilies, (B) Y-enriched TE subfamilies" – I think is a typo and B should be "Y-depleted TE subfamilies"

- File "Supp5" should be modified to have multiple columns.
