# Supplementary material for: Transposable element expression is associated with sex chromosome number in humans
Source: PLoS Genet. 2025 Jun 26;21(6):e1011668. doi: 10.1371/journal.pgen.1011668 (PMC12225981; doi:10.1371/journal.pgen.1011668)
Supplement: Attachment — Submitted filename: Response to reviewers round1_31_01_2025.pdf [file pgen.1011668.s011.pdf]

Plos Genetics general considerations

Authors' reply:
We gratefully thank the editor and reviewers for giving us the opportunity to improve our manuscript. We have taken all the reviewers' comments into account as far as possible. This also led us to update the abstract and authors' summary.

Please be aware that our data availability policy requires that all numerical data underlying graphs or summary statistics are included with the submission, and you will need to provide this upon resubmission if not already present. In addition, we do not permit the inclusion of phrases such as "data not shown" or "unpublished results" in manuscripts. All points should be backed up by data provided with the submission.

Authors' reply: This has been done.

While revising your submission, please upload your figure files to the Preflight Analysis and Conversion Engine (PACE) digital diagnostic tool. PACE helps ensure that figures meet PLOS requirements. To use PACE, you must first register as a user. Then, login and navigate to the UPLOAD tab, where you will find detailed instructions on how to use the tool. If you encounter any issues or have any questions when using PACE, please email us at figures@plos.org.

Round #1

All of the reviewers were enthusiastic about the study overall, but each had multiple concerns with the analyses and inclusion of appropriate controls and statistical comparisons. These will need to be addressed in a revised submission.

Reviewer's Responses to Questions

Reviewer #1:

In this manuscript, Teoli et al set out to test whether Y-linked TEs may contribute to the sex gap in longevity in humans, a phenomenon known as the "toxic Y" effect. They analyze two RNA-seq datasets to address this question: (1) expression profiles from blood of groups of patients with different sex chromosome complements (XX, XY, XXY, XYY) and (2) GTEx expression profiles from blood of XX females and XY males across different age groups. In the first analysis, they find a fairly consistent trend where a variety of TE subfamilies show increasing expression across the karyotype gradient XX, XY, XXY, and XYY. In the second analysis they find no difference in TE expression between XX and XY groups or between different age groups within sexes.

Unfortunately, after reading the manuscript, I am unsure of what can be concluded from the work presented here. The results from the first analysis are interesting but aren't directly related to the toxic Y/longevity hypothesis: the upregulation of TEs also occurs in XXY individuals and there is no connection to ageing in this analysis. On the other hand, although the second analysis does test for differences in TE expression across age groups, no significant differences are found.

This manuscript has already been reviewed through PCI Genomics, which I am not familiar with. I largely agree with the previous reviewers comments and the authors revisions, however I have additional feedback that hopefully may be useful.

(1) The authors frame their work as addressing whether Y-linked TE expression could contribute to differences in longevity between sexes but I don't think that these are the best datasets to test such a question. The GTEx analysis seems to be a negative result but there are so few samples in each age group it is unclear whether the lack of a difference in TE expression with age is real or just a result of limited power. I appreciate the authors acknowledgement of the limitations of their datasets, but I would encourage them to consider whether the toxic Y hypothesis is the best way to frame this work, especially because there also seems to be an effect on TE expression from having an extra X chromosome. Why not just frame this as a test of the effect of sex chromosome complement on TE expression?

Authors' reply: We thank the reviewer for this comment. We have revised the text to better frame it around the effects of sex chromosomes on TE expression.

Therefore, we have modified:

- The title to : "**Transposable element expression is associated with sex chromosome number in humans**"
- Some sentences in all the manuscript to reduce its orientation toward a toxic Y effect, emphasizing the effect of sex chromosomes rather than Y alone when appropriate (see, for instance, **lines 300-306 and 447-454**).

(2) The authors briefly mention another important genome-wide effect caused by the Y chromosome, which is that there is evidence that the Y can act as a "sink" for heterochromatin, resulting in the redistribution of heterochromatic marks from the autosomes onto the Y. If this is the case, autosomal genes that are normally silenced or lowly expressed in XX individuals should increase in expression along with Y chromosome dosage (and also potentially with an extra X chromosome as well depending upon whether the extra X is inactivated). It should be possible to test for this effect using the RNA-seq data from XX, XY, XXY, and XYY individuals.

Authors' reply: We have compared gene expression according to karyotype and we found that some genes carried by autosomes were differentially expressed between karyotypes (see **Table S2 and lines 174 to 187 in the main text**). We think it is more complicated than simply looking at which genes are upregulated in the presence of a supernumerary Y chromosome, because the expression of a usually silent gene may also be related to the regulation effect induced by a gene carried by a sex chromosome rather than by a sink effect. Moreover, the expression of a usually silent gene may regulate the expression of other genes and leads to their upregulation or downregulation. RNAseq data are probably not the best data to answer this kind of question (e.g. epigenetic data or chromatin conformation data would be better). Thus, to take into account the reviewer's comment, we added a section on the sink effect in the discussion section and indicated that it would be interesting to carry out new studies to evaluate it in humans (see **lines 495-502**).

(3) It is striking to me how consistent the pattern of TE upregulation is for the same individuals across multiple TE subfamilies. For example, in Fig 1, the 26 year-old XY male is an outlier in every panel. This could be real biology or an issue with normalization. There is no need to use DESeq normalization before the "Proportion of TE counts" expression calculation used by the authors. Their proportion measurement already accounts for differences in sequencing depth because it is a proportion, rather than simply a count of raw reads. I would encourage the authors to confirm that the patterns in Figures 1 & 2 are reproducible when calculating the proportion measurement from raw counts rather than DESeq normalized counts.

Authors' reply: We confirm that the patterns in **Figures 1 & 2** (as on **Figures S10 and S13**) are reproducible when calculating the proportion measurement from raw counts rather than DESeq normalized counts. Deseq2 generates a normalization factor specific to each individual. This same factor is applied to the counts of each sub-family of transposable elements and each gene, and similarly affects the numerator and denominator of our proportion calculation. Thus, raw TE counts / all raw counts (TEs and genes) is equivalent to normalized TE counts / all normalized counts (TEs and genes).

Minor comments:

Line 58: "increased TE activity": the authors should be careful to make clear that their results are only with respect to TE expression and not activity since they did not search for new TE insertions in their subjects.

Authors' reply: The reviewer is perfectly right, we have replaced "TE activity" by "TE expression" when appropriate.

The authors calculate the enrichment of various TE subfamilies on the Y chromosome, presumably using the hg38 genome assembly. They may want to consider redoing these calculations using the newer T2T human Y chromosome assembly, which likely contains many more TE copies that were missing from the version in hg38.

Authors' reply: Thanks for your comment.

We have retrieved the reference sequences of repeated elements based on the new T2T assembly here:

https://genome.ucsc.edu/cgi-bin/hgTables?hgsid=2434862507_pGnS3ljkYs9dSMzpQ4fFqtE9ALrR&clade=mammal&org=Human&db=hs1&hgta_group=allTracks&hgta_track=hub_3671779_t2tRepeatMasker&hgta_table=0&hgta_regionType=genome&position=chr9%3A145%2C458%2C455-145%2C495%2C201&hgta_outputType=primaryTable&hgta_outFileName=

output format = "sequence".

Then we determined the subfamilies of Y- or X-enriched transposable elements in the same way as described in the materials and methods section for the hg38 assembly. We did find some discrepancies: some transposable elements were enriched on the Y or X with the hg38 assembly and not with the T2T assembly, and vice versa. Some TE subfamilies (74) in the hg38 assembly were no longer found in the T2T assembly, probably due to a change of label or name (indicated as "NA").

n = 1246 TE subfamilies

	T2T: enriched Y	T2T: not enriched Y	T2T: NA
hg38: enriched Y	42	69	2
hg38: not enriched Y	227	834	72

	T2T: enriched X	T2T: not enriched X	T2T: NA
hg38: enriched X	140	83	18
hg38: not enriched X	65	884	56

For the 60 differentially expressed TEs in **Figures 2 and S12**, here's what we found:

	T2T: enriched Y	T2T: not enriched Y	T2T: NA
hg38: enriched Y	7	13	0
hg38: not enriched Y	3	33	4

	T2T: enriched X	T2T: not enriched X	T2T: NA
hg38: enriched X	2	5	3
hg38: not enriched X	1	48	1

To remain consistent, we have preferred not to indicate these new results in the article, since all the rest of the study is based on the hg38 assembly. Nevertheless, it would indeed be important to use the new T2T assembly for future studies in the field. Thus, we have mentioned it in the discussion (see line **547**).

The "proportion of TE counts" is a fairly conservative measure, especially when used for all TEs combined since the upregulation of some TE subfamilies will be masked by downregulation of other subfamilies. A paired Wilcoxon test would be much more sensitive if the goal is to test for an overall upregulation of a group of TE subfamilies. This would involve using paired expression values between karyotypes, for example, normalized counts of each TE subfamily from the XX group paired with their normalized counts from the XY group. The test calculates whether the median of the differences between the paired expression measurements differs significantly from zero.

Authors' reply: We thank the reviewer for this suggestion. We compared the median of log2 fold-change of normalized counts to zero in each pairwise karyotype comparison using a Wilcoxon test. This is equivalent to a paired Wilcoxon test comparing log2(normalized counts) in a first karyotype to the log2(normalized counts) in a second. We have added the new results obtained in **lines 273 to 279** and we have updated the **Fig. S11** .

Reviewer #2:

Review for "Transposable element expression with variation in sex chromosome number: insights

into a toxic Y effect on human longevity"

PLoS Genetics September 2024

Teoli et al. seek to answer whether Y chromosome copy number is associated with increased transposable element (TE) expression in humans. The authors perform gene expression profiling (RNA-seq) on whole blood samples from study participants with four different karyotypes: XX, XY, XXY, and

XYY, allowing them to measure the effect of Y chromosome dosage on gene and TE expression. Gene expression across the four groups is examined first. Despite clear separation on the PCA plot, XY vs XX groups show only a small number of differentially expressed (DE) genes. However, XXY vs XX and XYY vs XX show large numbers of differentially expressed genes, while XY vs XXY or XYY show a moderate number of differentially expressed genes. The authors then quantify all TEs and TE family/sub-family expression from the same RNA-seq dataset, using TETools. For the "all TEs" group, and all TE families except DNA transposons, expression levels seem to show an overall trend of increasing as Y chromosome copy number increases. However, the differences between karyotypes only reach statistical significance in certain comparisons and TE families. No TE family has significantly higher expression in the XY group compared to XX. For LTR and SVA TEs, XYY has significantly higher expression than the XY group. Several TE subfamilies are identified which do show statistically significant increases in XY vs XX, and XYY vs XY, which are primarily LTR and HERV subfamilies. As TE expression is thought to increase with age, the authors also measure TE expression in a separate, larger cohort of XX and XY subjects of ages 20-70 years old. Contrary to the authors initial hypothesis, old XY subjects do not have higher TE expression than old XX subjects, nor was there a significant change in overall TE ("all TEs") expression with age in either sex. In this second cohort, three TE sub-families showed higher expression in XY vs XX subjects across multiple age groups, while no sub-family showed higher expression in XX vs XY. The primary conclusion of the study is that the number of Y chromosomes is associated with increased TE expression, particularly within certain TE sub-families.

Overall, the study provides important and novel experimental insight into the effect of Y chromosome copy number on TE expression. To my knowledge, no prior similar studies have been conducted in human subjects with sex chromosome aneuploidies (though similar studies have been performed in Drosophila, which the authors discuss). The study is somewhat limited in scope, as all of the figures are generated from RNA-seq data from two datasets, one of which has a small sample size. Still, the authors analyze these datasets thoroughly, as seen in the extensive supplementary materials. The main finding that Y chromosome copy number is associated with higher TE expression in humans is convincing and should be of interest and value to the field. However, there are a number of concerns that should be addressed before I can recommend for publication. Thus, my overall recommendation is for the authors to address these concerns and re-submit.

Major concerns:

1. In general the writing is clear, but there are so many comparisons (often feeling somewhat random, e.g. focusing on XX vs XY one paragraph and XX vs XYY the next) and supplementary analyses, the main points can be easily lost or missed. The manuscript would benefit from editing to streamline and improve clarity. Specific things that would help include:

- **Establish and state the primary comparisons of interest for testing the main hypotheses of the study (I believe to be XY vs XX and XYY vs XY, but the authors should state clearly)**
- **Consistent presentation of comparisons (i.e. consistently discuss the same comparisons in the same order, e.g. XX vs XY, then XY vs XYY)**
- **The addition of conclusions to end of paragraphs in results section.**

Authors' reply: We thank the reviewer for this suggestion to improve clarity of our manuscript. We have stated the primary comparisons of interest for testing the main hypotheses of the study in the introduction section (**lines 147 to 150**). We have also reviewed the organization of the paragraphs (**lines 208 to 224, lines 226 to 249, lines 266 to 272, lines 273 to 279, lines 289 to 299**) in the results section to improve the constituency of the presentation of comparisons. Moreover, we have added some new conclusion sentences at the end of paragraphs (see l**ines 197 to 199, lines 221 to 224, lines 247 to 249**, **lines 300 to 306**, **lines 336-337, lines 358-359, lines 439 to 444**).

2. It seems a little strange that XX and XY groups separate well on PCA (Fig S2) but have very few differentially expressed genes that reach statistical significance between the two groups. Is this consistent with other blood RNA-seq studies?

Authors' reply: We agree with the reviewer. The discrepancy between the number of differentially expressed genes (**Table S2**) and the clear clustering of XY and XX individuals on PCA (**Fig S2**) is likely explained by the different construction approach. PCA is based on the expression of the 500 genes that explain the most variance (particularly those on the Y and X chromosomes), not on differentially expressed genes. The heatmaps **Fig S4** clearly show that some genes are expressed very differently in XY and XX individuals and these genes are sufficient to create a great distance between XY and XX individuals because of their important log2 fold change.

In blood RNAseq studies, it was reported a few number of differentially expressed genes between men and women (about 0.1% of genes) whereas men and women clustered in unsupervised clustering analysis based on gene expression (Guillén et al. 2014 - Analysis of gene expression profile for gender in human blood samples).

3. The manuscript repeatedly says the Y chromosome is enriched for TEs, but don't provide any sort of quantification for this. Please provide some type of numerical frame of reference for TE enrichment on Y chromosome. For example, see Erlandsson, Wilson, Paabo 2000 reference from the manuscripts reference list. Based on this information, what would be the predicted impact of Y chromosome copy number on % increase in TE expression?

Authors' reply: We have added the recent reference Rhie et al. 2023 showing that the human Y chromosome sequence (T2T-Y assembling) is represented by 85% of repeats (including 35.8% of TE). The Y chromosome is richer in repeat elements than the rest of the genome but is not richer in TE insertions. We think that Y toxic hypothesis in human may rely on the redistribution of heterochromatin mark ("sink effect") induced by the presence of Y chromosome (due to its richness in repeat sequences) and/or by the presence of more transpositionnally or transcriptionally active TE insertions on Y chromosome rather than the TE content of the Y chromosome. Therefore, we have reworded sentences indicated that Y chromosome is richer in TE content than other chromosomes and replaced transposable elements by repeat elements when relevant (see **lines 102 to 109 and 137 to 142** in the introduction**, lines 482 to 502** in the discussion).

4. Lines 216 and 421: the authors note twice that addition of an extra X chromosome (ie XXY) is also associated with higher TE levels, "probably/likely" due to "increased genomic material" and, consequently, TE load. What do the authors mean exactly by "increased genomic material?" Maybe I am misunderstanding this point, but I believe RNA-seq library prep generally uses a standardized amount of input RNA from all samples (it is not clear from the methods if the authors did this), which would correct for differences in genomic material. In addition, RNA-seq analysis is based on relative expression and thus, unless a spike-in is used, differences in expression based on initial input are lost during normalization. Could the authors clarify this point and the methods used for library prep?

Authors' reply: We thank the reviewer for pointing this out. We have rewritten the sentences referring to "genomic material", therefore the words "increased genomic material" do not appear anymore in the text. Furthermore, the reviewer is right : the same input of RNA was used for all individuals and libraries were normalized before sequencing as described in the protocol of the Illumina TruSeq Stranded Total RNA Lp Gold kit. We have added this precision in the **Materials and Methods section (lines 599 and 604-605)**.

5. Are there any other reasons besides "increased genomic material" that might explain why XXY are often found to have (a trend towards) higher TE expression than XY?

Authors' reply: We think the answer is yes. Like the Y chromosome, the X chromosome could induce a redistribution of heterochromatin marks promoting TE expression (sink effect) but to a lesser extent,

as observed in Drosophila (Brown et al. 2020. The Drosophila Y chromosome affects heterochromatin integrity genome--wide.). However, this point remains to be assessed in humans. We now discuss this point **lines 495 to 498**.

6. In relation to Figure 2 and S12, could the authors make a table for each main TE family, showing the number and percent of TE sub-families which were:

- **Significantly higher in XY vs XX**
- **Significantly higher in XYY vs XY**
- **Significantly lower in in XY vs XX**
- **Significantly lower in XYY vs XY**

This would provide helpful context to the results shown in Figure 2 and would be easier to digest than Figure S12.

Authors' reply: In the hope that we have correctly understood the reviewer's suggestion, we are now providing two new supplementary data (**Data S4** and **Data S5**). **Data S4** shows the classification of the differentially expressed TE subfamilies in each pairwise karyotype comparison. Then, we have calculated the number of TE subfamilies upregulated and downregulated per TE family, superfamily/order/class in all the pairwise karyotype comparisons in **Data S5**.

7. Only 1 (MER52D) of 3 TE sub-families found to be significantly higher in XY than XX from the second cohort in Figure 4 also show up as significantly higher in XY vs XX in Figure 2 and Figure S12. Can the authors comments on this – does it suggest a lack of agreement of consistency between datasets?

Authors' reply: We have added a comment on this point (discussed **lines 535 to 539**). We think mismatches between the GTEx datasets and gonosome aneuploidy dataset are due to :

- The technical and biological noises that would impact the GTEx datasets, noises that are minimized in the gonosome aneuploidy dataset (see **lines 533 to 536**).
- The differences in the GTEx datasets and gonosome aneuploidy dataset in the library preparation and sequencing : non-stranded polyA+ library and 75x2 paired-end sequencing in GTEx datasets versus stranded total RNAseq with ribodepletion and 151x2 paired-end sequencing in gonosome aneuploidy dataset (see **lines 599 to 610 and lines 613-614** in the **materials and methods**).
- The sequencing depth was also lower in the GTEx datasets than the gonosome aneuploidy dataset (see **line 607 and line 651** in the **materials and methods**).

8. Figure S14 shows roughly the same pattern for TE expression across the 4 karyotypes regardless of whether the TE sub-families are enriched, depleted, not enriched or depleted, or not present at all on Y or X chromosomes. Could the authors discuss whether this weakens the central conclusion that increased TE expression with increasing Y copy number is due to a high number of TEs on the Y chromosome?

Authors' reply: We do not think this data weakens our conclusions. We simply conclude that the Y chromosome may have an effect on the expression of transposable elements, whether Y-carried or not. Although this point remains to be verified by an expression study of the TEs at locus level (discussed in **lines 542 to 549**). Furthermore, our analysis cannot rule out the possibility that TE copies on the Y chromosome are transcribed more actively than those on other chromosomes. A TE subfamily may appear not to be Y-enriched if it has more copies on other chromosomes than on the Y chromosome; however, a single TE copy on the Y chromosome could be transcribed at a much higher level than others, contributing to differences in TE expression between karyotypes.

9. Line 276 "…the proportion of copies of this TE subfamily carried by the Y chromosome increased, was also more pronounced in the Y-enriched TE subfamily group than in the not Y-enriched TE subfamily group (Fig. S13B)." This sentence is confusing and could be written more clearly. Given the accompanying Figure S13B, I am not sure what the point or conclusion is.

Authors' reply: We feel that this point is not very important, and could lead to confusion. We have therefore decided to delete this figure and the sentences referring to it.

10. Line 306 "upstream regions" should be defined, i.e. in base pairs from transcription start site.

Authors' reply: We have defined upstream regions **line 328 ("3 kb from the transcription start site")**.

Minor points:

• The discussion would benefit from adding a reference to De Cecco et al., 2019 (Nature volume 566, pages73–78) when discussing mechanisms by which increased TE expression can be harmful without transposition. Gorbunova et al., 2021 (Nature volume 596, pages43–53) may also be worth adding.

Authors' reply: We thank the reviewer for the suggestion. We have added these references in **lines 120 and 575**.

• Line 166 of supplementary material for Fig S14 "(A) Y-enriched TE subfamilies, (B) Y-enriched TE subfamilies" – I think is a typo and B should be "Y-depleted TE subfamilies"

Authors' reply: The reviewer is absolutely right. Thank you very much for pointing this out. We have made the correction.

• File "Supp5" should be modified to have multiple columns.

Authors' reply : We have added the same files in excel format.

Reviewer #3:

The manuscript "Transposable element expression with variation in sex chromosome number: insights into a toxic Y effect on human longevity" explores what the role of TE expression might be in sex differences in aging.

This is an interesting unsolved problem in aging research: why do males and females age at different rates (or more specifically, why is lifespan different across sexes)? One intriguing possibility is that the sex chromosomes themselves harbor TEs that, when expressed during age epigenetic/transcriptional dysregulation, may be deleterious—with preferential effects on one sex. There certainly seems to be ample evidence of this in flies, which the authors hope to build upon.

I'm broadly enthusiastic about this manuscript, I think it will be an important finding for the field of TE and aging, and hope that the authors can improve it to the (admittedly, very high) standard of PLoS Genetics.

Here are my major concerns:

1. The authors mention that the human Y chromosome is enriched for TE content compared to other chromosomes, and that this may provide a conceptual basis for male-specific toxic TE effect. However, I would like to point out that the human X chromosome is also extremely enriched for LINE L1 retrotransposons (Bailey, et al. PNAS 2000). The X chromosome is a large chromosome, with 1000 genes. Based on these other arguments, it might seem like females (46 XX, 47XXX) or 47XXY males might have a toxic X effect—although admittedly, only one X should be active in 46XX, 47XXX, or

47XXY individuals if they have stable X chromosome inactivation. Additionally, stating that the Y chromosome is "enriched for TE" should be backed up with some analysis of the age of TE on the Y or the intactness (ability to be transcribed etc)—since not just the presence of TE on the Y, but their ability to be made into RNA through transcription is the mechanistic basis of the toxic Y model. To be fair to the reader, the authors should discuss this alternative possibility (X chromosome/TE toxicity) and explain if they think this is not a viable hypothesis.

Authors' reply: We agree with the reviewer's comment. According to the last human genome assembly (T2T), the Y chromosome is extremely rich in repeat elements (not necessarily transposable elements) and few TE copies carried by the Y chromosome are complete copies. However, incomplete TE copies could also be expressed, and as noted in **lines 118 to 120 and lines 570 to 575**, the "toxic" effects of TEs may arise through mechanisms other than transposition.

We think that the sink effect induced by the Y repeats may explain our results suggesting a higher expression of TE in XYY individuals. Perhaps a sink effect is also induced by the X chromosome but to a lesser extent since the supernumerary X chromosomes are inactivated in humans. We now discuss this point in the discussion part (see **lines 495 to 502**). The sink effect remains to be studied in humans.

2. Mosaic Y chromosome loss in white blood cells is estimated to occur in 20% of men (Thompson, et al. Nature Genetics 2019). Is there any way that the authors can ensure that the 46XY, 47XYY, or 47XXY males do not have mosaic Y loss? I would imagine that this phenomenon would complicate their study.

Authors' reply: The reviewer is right. The Y chromosome loss may be a conservative bias in our study. However, in the gonosome aneuploidy dataset, the karyotype was homogeneous (or nearly so), as indicated in Materials and Methods, **lines 584 to 586**.

In the GTEx datasets, individuals' karyotype was not available. We observed a trend towards decreased expression of Y genes, which we used as a proxy to assess Y chromosome loss, when the age increased. This trend was not significant. Nevertheless, we added a sentence in the results section (**see lines 394 to 400**) and a new figure in supplementary materials (**see Fig S17**).

3. A major concern for this work is that the RNA-seq that is the foundation of the conclusions is based on bulk RNA expression from white blood cells. If there are sex-specific differences in the type of white blood cells, and each type of cell has its own transcriptional profile, and each TE family can respond to cell-type specific transcription factors, then the TE expression differences they see with age/sex are not a reflection of the toxic Y per se, but rather from different cell compositions of blood in age/sex variable manner. There is published work showing that white blood cell composition is slightly different in males and females (Bongen, et al. Cell Reports 2019). A nice piece of data that argues against this concern is that the authors find that the TE families that are upregulated in aged Y chromosome containing individuals are found as TEs enriched on the Y chromosome. This specific concern could be addressed by mining the publicly available scRNAseq datasets from human white blood cells. If we can now compare specific cell types vs specific cell type using the scRNAseq data, can we pinpoint if there is a single cell type that contributes this TE upregulation in Y-chromosome containing individuals? Or do all cells in the blood show similar TE dysregulation? This type of analysis is likely outside of the scope of the current work, but I would encourage the authors to consider utilizing these publicly available data for follow-up studies. They could make a substantial contribution in this area using their analysis pipeline with scRNAseq datasets.

Authors' reply: We gratefully thank the reviewer for this comment which we will consider in our future work. Furthermore, we added this suggestion in the discussion section as a perspective for a future work (see **lines 542 to 544**).
