# Supplementary material for: Transposable element expression is associated with sex chromosome number in humans
Source: PLoS Genet. 2025 Jun 26;21(6):e1011668. doi: 10.1371/journal.pgen.1011668 (PMC12225981; doi:10.1371/journal.pgen.1011668)
Supplement: Attachment — Submitted filename: Reply to reviewers round2_19_03_2025.pdf [file pgen.1011668.s012.pdf]

Plos Genetics general considerations

Dear Dr. Teoli,

Thank you for submitting your manuscript to PLOS Genetics. After careful consideration, we feel that it has merit but does not fully meet PLOS Genetics's publication criteria as it currently stands. Therefore, we invite you to submit a revised version of the manuscript that addresses the points raised during the review process.

Please submit your revised manuscript within **30 days Apr 16 2025 11:59PM**. If you will need more time than this to complete your revisions, please reply to this message or contact the journal office at plosgenetics@plos.org. When you're ready to submit your revision, log on to https://www.editorialmanager.com/pgenetics/ and select the 'Submissions Needing Revision' folder to locate your manuscript file.

Please include the following items when submitting your revised manuscript:

*** A rebuttal letter that responds to each point raised by the editor and reviewer(s). You should upload this letter as a separate file labeled 'Response to Reviewers'. This file does not need to include responses to formatting updates and technical items listed in the 'Journal Requirements' section below.**
*** A marked-up copy of your manuscript that highlights changes made to the original version. You should upload this as a separate file labeled 'Revised Manuscript with Track Changes'.**
*** An unmarked version of your revised paper without tracked changes. You should upload this as a separate file labeled 'Manuscript'.**

If you would like to make changes to your financial disclosure, competing interests statement, or data availability statement, please make these updates within the submission form at the time of resubmission. Guidelines for resubmitting your figure files are available below the reviewer comments at the end of this letter.

We look forward to receiving your revised manuscript.

Kind regards,

Giorgio Sirugo
Section Editor
PLOS Genetics

Aimée Dudley
Editor-in-Chief
PLOS Genetics

Anne Goriely
Editor-in-Chief
PLOS Genetics

Additional Editor Comments :

Please address comments by Reviewer 2.

Journal Requirements:

1) Thank you for stating "Fastq files of the gonosome aneuploidy dataset generated in this study were submitted to the European Genome-phenome Archive (EGA ID of the study: EGAS00001007462). " Please note that, though access restrictions are acceptable now, your entire minimal dataset will need to be made freely accessible if your manuscript is accepted for publication. This policy applies to all data except where public deposition would breach compliance with the protocol approved by your research ethics board. If you are unable to adhere to our open data policy, please kindly revise your statement to explain your reasoning and we will seek the editor's input on an exemption."

Round #2 - Reviewers' comments:

Reviewer's Responses to Questions

Authors' reply:
We would like to thank the reviewers for their positive feedback on the changes we have made. We would like to thank reviewer 2 for these new proposed modifications, which will further improve the clarity of our manuscript.

Reviewer #1:

The authors have done a nice job addressing my previous comments, I have no further suggestions.

Reviewer #2:

Overall, the authors have done an excellent job responding to my concerns, both in their response letter and in their revisions to the manuscript. In particular, the authors did a good job of stating the primary comparisons of interest, and reporting results for these comparisons in a consistent manner throughout the manuscript. In addition, "Data S5" is a very helpful addition (particularly columns E and F), which makes it much easier to see results for TE subfamilies across all comparisons. The clarity and accuracy of the writing has also improved. There are many supplemental data figures and tables contained within the manuscript, which can be difficult to navigate and distinguish between, but these data are well organized and well described and should be a useful resource to other researchers in the field. The central finding that sex chromosomes, particularly the Y chromosome, might be associated with TE expression does appear to be supported by the data.
Overall I now support accepting this article for publication in PLoS Genetics. However, there are a few minor writing issues I would like to see resolved.

Minor concerns:

1. Lines 243-246 "In humans, only SVA, HERVK, AluS, L1, and AluY are known to be transcriptionally active…" This is a little confusing and could use a transition. In the previous paragraphs, the authors were focusing on "five TE classes/orders/superfamilies (LTR order), SVA superfamily, SINE superfamily, LINE superfamily, and DNA transposon class)…" and now appear to be focusing on specific families.

Authors' reply: We have included the requested modifications.

2. Line 266 is the beginning of a new results section (ie now referring to Figure 2) and the first sentence begins with "This time…" which does not really make sense in this context. The phrase "this time" typically follows some context of the "previous time."

Authors' reply: We have included the requested modifications.

3. Lines 273-279: why are results now being described in terms of log2 fold change? Nowhere else are results described in this manner. This section appears to have been added in revision. It sort of comes out of nowhere, and could use an introduction/explanation.

Authors' reply: We have included the requested modifications.

4. Line 280 starts with "we finally" but this is not the "final" result of this section. The next paragraph begins with "furthermore."

Authors' reply: We have included the requested modifications.

5. In the authors reply to comment 3 in my first review (ie reviewer 2, comment 3), they mention "The Y chromosome is richer in repeat elements than the rest of the genome but is not richer in TE insertions." This is an important point, and the authors did add it to the manuscript (line 488-489 of discussion), but it would be helpful to state this earlier in the manuscript, ie in the introduction or results.

Authors' reply: We have included the requested modifications in the introduction section.

6. In the authors reply to comment 5 in my first review (ie reviewer 2, comment 5), they mention "Like the Y chromosome, the X chromosome could induce a redistribution of heterochromatin marks promoting TE expression (sink effect) but to a lesser extent, as observed in Drosophila." The discussion states (line 497-498) "In Drosophila, the sink effect induced by the Y chromosome was more important than those induced by the X chromosome." This is a fairly vague description of the X chromosome sink effect. Given this concept is an important point for the conclusions of this paper, the authors should add more detail (and references, if possible) about what is know about the X chromosome acting to redistribute heterochromatin marks (sink effect), and thus possibly promoting TE expression.

Authors' reply: We have reworded this part with more detail.

7. Also, on line 496 of discussion, it would be helpful to add a little more description of the sink effect. How is heterochromatin redistributed to the Y chromosome and how does this affect other chromosomes?

Authors' reply: We have reworded this part with more detail.

Reviewer #3:

The authors have sufficiently addressed my concerns.

Have all data underlying the figures and results presented in the manuscript been provided? Large-scale datasets should be made available via a public repository as described in the PLOS Genetics data availability policy, and numerical data that underlies graphs or summary statistics should be provided in spreadsheet form as supporting information.

Reviewer #1: Yes

Reviewer #2: Yes

Reviewer #3: Yes

PLOS authors have the option to publish the peer review history of their article (what does this mean?). If published, this will include your full peer review and any attached files.

If you choose “no”, your identity will remain anonymous but your review may still be made public.

Do you want your identity to be public for this peer review? For information about this choice, including consent withdrawal, please see our Privacy Policy.

Reviewer #1: No

Reviewer #2: No

Reviewer #3: No

[NOTE: If reviewer comments were submitted as an attachment file, they will be attached to this email and accessible via the submission site. Please log into your account, locate the manuscript record, and check for the action link "View Attachments". If this link does not appear, there are no attachment files.]

Figure resubmission:

While revising your submission, please upload your figure files to the Preflight Analysis and Conversion Engine (PACE) digital diagnostic tool, https://pacev2.apexcovantage.com/. PACE helps ensure that figures meet PLOS requirements. To use PACE, you must first register as a user. Registration is free. Then, login and navigate to the UPLOAD tab, where you will find detailed instructions on how to use the tool. If you encounter any issues or have any questions when using PACE, please email PLOS at figures@plos.org. Please note that Supporting Information files do not need this step. If there are other versions of figure files still present in your submission file inventory at resubmission, please replace them with the PACE-processed versions.

Reproducibility:

To enhance the reproducibility of your results, we recommend that authors deposit laboratory protocols in protocols.io, where a protocol can be assigned its own identifier (DOI) such that it can be cited independently in the future. Additionally, PLOS ONE offers an option to publish peer-reviewed clinical study protocols. Read more information on sharing protocols at https://plos.org/protocols?utm_medium=editorial-email&utm_source=authorletters&utm_campaign=protocols
